# Topical Polyherbal Phytopreparation Reduces Gingival Inflammation: Evidence from a Randomized Controlled Clinical Study Supported by In Silico Analysis

**DOI:** 10.3390/ph19030398

**Published:** 2026-02-28

**Authors:** Milica Petrović, Ljiljana Kesić, Jovana Veselinović, Jelena Popović, Nikola Živković, Bojana Miladinović, Miloš S. Jovanović, Branislava Stojković, Simona Stojanović, Dušanka Kitić

**Affiliations:** 1 Department of Oral Medicine and Periodontology, Dental Clinic, Faculty of Medicine, University of Niš, 18108 Niš, Serbia; 2 Faculty of Pharmacy Novi Sad, University of Business Academy, 21000 Novi Sad, Serbia; jovana.veselinovic@ffns.ac.rs; 3 Department of Restorative Dentistry and Endodontics, Dental Clinic, Faculty of Medicine, University of Niš, 18108 Niš, Serbia; jelena.popovic@medfak.ni.ac.rs; 4 Department of Pathology, Institute of Pathology, Clinical Center, Faculty of Medicine, University of Niš, 18108 Niš, Serbia; nikola.zivkovic@medfak.ni.ac.rs; 5 Department of Pharmacy, Faculty of Medicine, University of Niš, 18108 Niš, Serbia; bojana.miladinovic@medfak.ni.ac.rs (B.M.); milos.jovanovic@medfak.ni.ac.rs (M.S.J.); dusanka.kitic@medfak.ni.ac.rs (D.K.); 6 Department of Preventive and Pediatric Dentistry, Dental Clinic, Faculty of Medicine, University of Niš, 18108 Niš, Serbia; branislava.stojkovic@medfak.ni.ac.rs; 7 Department of Oral Surgery, Dental Clinic, Faculty of Medicine, University of Niš, 18108 Niš, Serbia; simona.stojanovic@medfak.ni.ac.rs

**Keywords:** periodontitis, scaling and root planing, phytotherapy, cytomorphometry, molecular docking, in silico analysis

## Abstract

**Background**: Numerous plant-derived products have shown notable potential in preclinical studies and traditional use for the management of periodontitis, although clinical studies validating their efficacy remain scarce. The present study investigated the efficacy of a polyherbal phytopreparation as an adjunctive therapy to scaling and root planing (SRP) in patients with periodontitis, and further examined its underlying mechanisms of action, pharmacokinetic behavior, and toxicological profile using in silico approaches. **Methods**: Eighty patients with moderate periodontitis (stage II, grade A) were randomly assigned to two groups: a control group (*n* = 40) treated with SRP alone, and an experimental group (*n* = 40) receiving SRP followed by topical phytotherapeutic treatment with the polyherbal Tinctura paradentoica^®^. Efficacy was evaluated using the gingival index, periodontal pocket depth, and cytomorphometric analysis of gingival cells before treatment and one month after. The in silico analysis, guided by HPLC profiling, included MolDock-based docking to assess interactions of bioactive compounds with cyclooxygenase isoforms COX-1 and COX-2 as anti-inflammatory targets, and evaluation of their pharmacokinetic and toxicity properties (ADME/Tox) using SwissADME, ProTox-3.0, and pkCSM. **Results**: Compared with SRP treatment, the experimental treatment significantly reduced the gingival index and periodontal pocket depth (*p* < 0.05), as well as the assessed cytomorphometric parameters (nuclear area, perimeter, and Feret’s diameter values) (*p* < 0.001). Rerank analysis revealed van der Waals-driven isoform selectivity: compact phenolic acids and aglycones favored COX-1, whereas bulky glycosides (e.g., rutin, narcissoside) were optimized for COX-2, with luteolin-7-*O*-glucoside showing near-balanced engagement. The ADME/Tox analysis indicated generally favorable pharmacokinetic and safety characteristics of phenolic compounds from the phytopreparation, including low systemic absorption and no predicted mutagenicity or skin sensitization potential. **Conclusions**: The topical application of the polyherbal phytopreparation demonstrated significant potential to enhance the efficacy of conventional SRP therapy by promoting the regression of gingival inflammation in patients with moderate periodontitis, further supported by in silico findings.

## 1. Introduction

Over the last ten years, fundamental research in periodontology has led to transformative shifts in how we perceive and understand the mechanisms behind periodontal tissue destruction. Advances in molecular science have shed light on the roles of genetics, environmental influences, and lifestyle choices in shaping biofilm composition and modulating the immune-inflammatory response, ultimately leading to the diverse biological condition known as periodontitis. This concept highlights a true symbiotic relationship among microbial communities and between those microbes and the host’s immune system [1]. In periodontitis, inflammation and microbial dysbiosis occur, which can lead to compromised barrier function of the gingival epithelia [2].

The inflammatory process in periodontitis triggers a cascade of immune and cellular responses that profoundly alter the cytomorphology of gingival epithelial cells. Pro-inflammatory cytokines, including interleukin-1*β*, interleukin-6, and tumor necrosis factor-*α*, stimulate epithelial cell stress, apoptosis, and accelerated turnover, resulting in nuclear enlargement, cytoplasmic shrinkage, and an increased nucleus-to-cytoplasm ratio [3,4]. These cytomorphometric changes reflect the severity of local inflammation and can serve as early microscopic indicators of periodontal tissue destruction and disease progression [5].

Cyclooxygenase (COX)-mediated prostaglandin biosynthesis plays a central role in gingival inflammation, making the COX isoforms, COX-1 (constitutively expressed) and particularly COX-2 (inducible), critical molecular targets for designing more effective therapeutic strategies in periodontitis management. Namely, while COX-1 is constitutively expressed in numerous tissues and essential for normal cellular function, COX-2 is an inducible enzyme whose expression is markedly increased in inflamed tissue. The regulation of COX expression may provide critical insights into disease mechanisms and serve as a basis for developing innovative diagnostic and therapeutic strategies for various oral conditions [6]. This elevated COX-2 expression in gingival tissues, evidenced in patients with periodontitis, is considered to contribute to disease pathogenesis. Accordingly, the application of selective COX-2 inhibitors with distinct selectivity profiles may provide protection against periodontitis progression, including inflammation and alveolar bone loss [7,8].

Chlorhexidine gluconate remains the most commonly employed oral antiseptic and antiplaque agent. However, prolonged use of this agent, as well as other synthetic compounds such as metronidazole and cetylpyridinium, has been associated with various adverse effects, including allergic reactions, unpleasant aftertaste, and tongue numbness [9,10]. Given the chronic and multifactorial nature of periodontitis, where microbial dysbiosis drives host inflammation, agents with combined antimicrobial and anti-inflammatory activity, while limiting long-term adverse effects, could markedly improve treatment outcomes. Such a multitarget therapeutic approach can be achieved through plant-derived preparations, given the complexity of their chemical composition and the consequent pharmacological activity mediated by multiple mechanisms [10,11,12]. This multitarget potential could be further enhanced through the formulation of polyherbal phytopreparations, allowing the selection of constituents to adjust the spectrum of bioactivities and optimize therapeutic outcomes [9,13]. Beyond their complex bioactivity, plant-derived preparations are often preferred over conventional drugs due to their favorable safety profile, lower therapy costs, biocompatibility, and minimal environmental impact [10]. Plant polyphenols have been shown to interfere with several pathogenic components through their anti-inflammatory, antimicrobial, and antioxidant properties, thereby representing promising therapeutic agents for periodontitis within such multitarget strategies [14]. Evidence from preclinical studies suggests that stilbenes, flavonoids, and proanthocyanidins are three main classes of plant polyphenols that exhibit activity against periodontitis, caries, and candidiasis [13]. Despite the recognized potential of phytopreparations in the management of periodontitis, robust evidence from randomized clinical studies remains limited.

Following our previous study on the topical application of the polyherbal phytopreparation (Tinctura paradentoica^®^), which demonstrated clinical efficacy as an adjuvant therapy for periodontitis through its impact on anaerobic bacteria from the “red complex” [15], the present study aimed to further evaluate its potential through the assessment of gingival index, periodontal pocket depth, and cytomorphometric features of gingival cells. Additionally, to gain deeper insight into the potential mechanisms of action of its bioactive compounds and their pharmacokinetic and toxicological properties, an HPLC-guided in silico analysis, including molecular docking and ADME/Tox profiling, was performed. To the best of our knowledge, this is the first study to apply an integrated approach, where clinical efficacy validation of a phytopreparation in periodontitis was supported by in silico analysis.

## 2. Results

This randomized controlled clinical study enrolled 80 participants, randomly assigned to two groups (Table 1). The control group (*n* = 40) received scaling and root planing (SRP) periodontal therapy, and the experimental group (*n* = 40) received phytotherapeutic agents in combination with SRP periodontal therapy.

### 2.1. Clinical Examination

The Gingival index (GI) values were significantly reduced in both the control and experimental groups after the fifth treatment (Wilcoxon signed-rank test, *p* < 0.001). However, one-month post-therapy, the GI values in the SRP group were significantly higher than those in the experimental group (Mann–Whitney test, *p* < 0.05). In the experimental group, the GI index showed significant reductions both after the fifth treatment (*p* < 0.01) and one month after therapy initiation (*p* < 0.05) when compared to the control (SRP) group (Mann–Whitney test). Statistically significant decreases in periodontal pocket depth (PPD) values were observed one month after therapy in both groups (Wilcoxon signed-rank test, *p* < 0.001). Furthermore, a significant difference was noted between the groups one month after therapy, with the experimental group showing a greater reduction in PPD values compared to the control (SRP) group (Mann–Whitney test, *p* < 0.001; Table 2). Notably, no side effects were observed in treated patients in both groups during the monitored period. At one month, the between-group difference in PPD, although statistically significant, corresponded to a small effect size (rank-biserial correlation r₍_rb_₎ = 0.12, 95% CI: 0.01–0.24). In contrast, the between-group reduction in the gingival index showed a larger effect (r₍_rb_₎ = 0.26, 95% CI: 0.10–0.40), indicating a more pronounced adjunctive benefit on gingival inflammation. After Holm–Bonferroni adjustment for the primary (GI) and key secondary (PPD) endpoints, the between-group differences remained statistically significant.

### 2.2. Cytomorphometric Evaluation

The values of nuclear area, perimeter, Feret’s diameter and integrated optical density were significantly lower (*p* < 0.001) in the experimental group compared to the control (SRP) group, a month after treatment. However, the values of the nuclear roundness and circularity did not change in the control group, but increased in the experimental group (*p* < 0.001) (Table 3).

### 2.3. Molecular Docking Evaluation

The complete docking readout for the HPLC-identified constituents against the cyclooxygenase isoforms is provided in Table 4 and Table 5. For each ligand, both the MolDock Score and the Rerank Score are provided together with their per-term energy components—steric/Lennard-Jones van der Waals and hydrogen-bond contributions—all expressed in kcal/mol. More negative values indicate stronger predicted binding.

**Figure 1:**
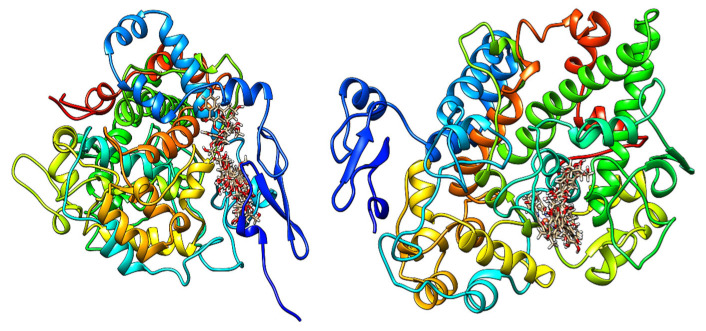
The best calculated poses for all studied molecules within the active site of human COX-1 (**left**) and COX-2 (**right**).

On COX-1, ranking by the MolDock Score showed the following order: hyperoside (−210.204 kcal/mol), narcissoside (−200.646 kcal/mol), rutin (−197.442 kcal/mol), luteolin-7-*O*-glucoside (−193.250 kcal/mol), and rosmarinic acid (−190.606 kcal/mol). On COX-2, the strongest MolDock Scores were observed for narcissoside (−212.057 kcal/mol), rutin (−210.146 kcal/mol), luteolin-7-*O*-glucoside (−199.463 kcal/mol), and hyperoside (−180.793 kcal/mol). When ranked by Rerank Score, the following ligands led on COX-1: hyperoside (−146.114 kcal/mol), chlorogenic acid (−135.500 kcal/mol), luteolin-7-*O*-glucoside (−135.262 kcal/mol), and rosmarinic acid (−131.799 kcal/mol). On COX-2, the top Rerank Scores were: narcissoside (−138.733 kcal/mol), rutin (−137.017 kcal/mol), luteolin-7-*O*-glucoside (−132.053 kcal/mol), and chlorogenic acid (−118.126 kcal/mol). Van der Waals (VdW) terms were positive for rutin (+40.034 kcal/mol) and vitexin (+84.202 kcal/mol) on COX-1, but negative on COX-2 (rutin −41.118 kcal/mol; narcissoside −42.024 kcal/mol). Hydrogen-bond contributions were substantial for several ligands, including rosmarinic acid on COX-1 (−29.293 kcal/mol) and chlorogenic acid on COX-2 (−24.802 kcal/mol). Rerank-based selectivity (Δ = COX-2 Rerank − COX-1 Rerank) showed COX-2 preference for rutin (Δ ≈ −49.7 kcal/mol) and narcissoside (Δ ≈ −34.2 kcal/mol), and COX-1 preference for hyperoside (Δ ≈ +34.2 kcal/mol), chlorogenic acid (+17.4 kcal/mol), rosmarinic acid (+15.5 kcal/mol), and isorhamnetin (+13.2 kcal/mol). Luteolin-7-*O*-glucoside showed near balance (Δ ≈ +3.2 kcal/mol). The best poses of studied ligands inside human COX-1 and COX-2 are presented in Figure 1. The 2D interaction maps for COX-1 (Figures S1–S9) and COX-2 (Figures S10–S18) are provided in the Supporting Information.

### 2.4. ADME/Tox Properties

The in silico predicted physicochemical properties, drug-likeness, ADME pharmacokinetics, and toxicity parameters of the tested phenolic compounds are presented in Table 6, Table 7 and Table 8. All compounds exhibited predicted molecular masses below 500 g/mol, except for narcissoside (624.54 g/mol). Based on the log S scale, water solubility was classified as soluble (log S between −4 and −2) or moderately soluble (log S between −6 and −4). The predicted consensus log Po/w values ranged from −0.39 for chlorogenic acid to 1.65 for isorhamnetin, indicating slightly hydrophilic to moderately lipophilic properties. Although less relevant for local applications, drug-likeness assessments showed that most compounds complied with Lipinski’s rule, except for narcissoside, luteolin-7-*O*-glucoside, and hyperoside.

ADME profiling indicated low gastrointestinal absorption for most compounds, except for gallic acid and isorhamnetin, while all were predicted to be unable to cross the BBB. Together with the highly negative skin permeation log Kp values (ranging from −10.12 to −6.82), these results suggest a potential for local retention and low systemic exposure of the tested compounds, supporting their suitability for topical application. Most compounds are not predicted to be P-gp substrates (except narcissoside and luteolin-7-*O*-glucoside) or CYP inhibitors (except gallic acid for CYP3A4 and isorhamnetin for CYP1A2, CYP2D6, and CYP3A4), indicating a low potential for drug–drug interactions via these pathways.

Regarding toxicity, most compounds were predicted to have an LD_50_ value of 5000 mg/kg, classifying them in class 5, except for vitexin (832 mg/kg) and gallic acid (2000 mg/kg), which were classified in class 4. Most compounds were predicted to be inactive for hepatotoxicity, neurotoxicity, and cardiotoxicity, whereas activity was predicted for nephrotoxicity and respiratory toxicity. Notably, none of the tested compounds were predicted to be skin sensitizers, which can be tentatively extrapolated as a low risk of local irritation when applied topically to gingival tissue. Furthermore, none of the compounds were predicted to be mutagenic in the AMES toxicity test.

## 3. Discussion

Periodontitis is a chronic inflammatory disease affecting nearly 60% of the global population, and the usage of local drug delivery systems and natural therapeutic agents may serve as promising adjuncts in the management of periodontitis, both in patients with and without systemic conditions such as cardiovascular disease and diabetes [16].

Phytotherapeutic agents have emerged as promising adjuncts in the treatment and prevention of periodontal and gingival diseases. Therefore, we applied Tinctura paradentoica*^®,^* and we observed cell morphology and clinical changes in the gingival tissue. The components of this solution were: marigold, yarrow, oregano, knotgrass, sage, tormentil tincture, and peppermint essential oil. The chemical composition of the investigated phytopreparation, determined previously by HPLC-DAD analysis [15], indicates a high content of phenolic compounds, particularly phenolic acids and flavonoids. The quantified polyphenolic compounds include: chlorogenic acid (162.85 ± 4.12 µg/mL), rutin (52.59 ± 1.39 µg/mL), gallic acid (11.12 ± 0.23 µg/mL), rosmarinic acid (1102.79 ± 21.56 µg/mL), isorhamnetin (24.17 ± 0.49 µg/mL), narcissoside (227.34 ± 3.78 µg/mL), vitexin (73.17 ± 2.15), luteolin-7-*O*-glucoside (358.06 ± 5.64 µg/mL), and hyperoside (19.89 ± 0.33 µg/mL). Owing to their broad biological activity and generally good safety profile, plant-derived materials can enhance the effectiveness of standard periodontal therapy while reducing the burden of intensive treatment approaches. As complex mixtures of different compounds, plant materials may exert their activity through multiple mechanisms and act as multi-target agents [10]. In line with our findings, numerous studies indicate that their anti-inflammatory activity represents one of the central mechanisms underlying their adjunctive effects during scaling and root planing therapy. Beyond anti-inflammatory effects, their efficacy in periodontal diseases is further supported by antimicrobial, antioxidant, and astringent activities [10,12,17]. Evidence suggests that, compared to conventional agents such as chlorhexidine or antibiotic gels, extracts from plants such as *Camellia sinensis*, *Punica granatum*, *Zingiber officinale*, and *Rosmarinus officinalis* provide comparable, though not superior, efficacy [12].

As shown in Table 2, both groups exhibited significant reductions in GI shortly after treatment and at one month, reflecting the dominant effect of scaling and root planing. However, the experimental group demonstrated significantly lower GI values than the SRP-only group at both post-treatment time points, indicating an additional anti-inflammatory effect of the phytopreparation. PPD also decreased significantly in both groups after one month, with a slightly greater reduction in the experimental group, suggesting a modest adjunctive benefit.

Scaling and root planing is the gold standard of non-surgical periodontal therapy. The use of adjunctive topical agents in our study follows the approach of our previous research, one month after therapy initiation, the intervention group receiving Tinctura paradentoica*^®^* showed significantly greater reductions in plaque index and bleeding on probing (*p* < 0.05) as well as a more pronounced gain in clinical attachment level (*p* < 0.001), indicating an additional benefit of the phytotherapeutic adjunct [15]. A growing body of evidence shows that herbal formulations, including *Aloe vera*, curcumin, green tea, guava leaf extract and *Triphala*, significantly improve gingival inflammation and bleeding, as well as the values of the GI and PPD decreased, while avoiding the adverse effects associated with chlorhexidine [18,19,20,21]. These findings support the rationale for using phytotherapeutic agents as safe and effective adjuncts to conventional periodontal treatment.

Although the between-group difference in PPD at one month was numerically small (0.08 mm), the rank-biserial correlation indicated a real but small effect of adjunct phytotherapy. This finding is consistent with the clinical context of Stage II, Grade A periodontitis, where most early pocket reduction is driven by scaling and root planing. This represents one of the observed outcomes and should be interpreted in the context of the overall clinical findings. Importantly, the larger effect size observed for the gingival index and the marked cytomorphometric improvements suggest that the phytotherapeutic agent primarily exerts its benefit through anti-inflammatory and tissue-modulating mechanisms rather than through short-term pocket depth reduction alone.

Cytology plays an important role in evaluating inflammatory conditions within the oral cavity. However, quantifying cellular structures and their components remains a significant methodological challenge for numerous investigators [5,22,23,24]. The gingival epithelium, composed of stratified squamous cells, undergoes continuous desquamation—a process influenced by the mitotic activity of basal epithelial cells, intracellular enzymatic activity, and external mechanical stimuli [22]. Only a limited number of studies have utilized exfoliative cytology to assess alterations associated with gingival inflammation and periodontal disease.

Oral exfoliative cytology is a simple, noninvasive, and atraumatic technique that involves the microscopic evaluation of cells harvested from the oral mucosal surface. Cytomorphometric analysis, in particular, is distinctive in its ability to identify cellular alterations at a subclinical level, thereby facilitating the early detection of pathological conditions and treatment effects [23].

The results of the present study reveal a significant decrease in the values of nuclear area, perimeter, integrated optical density and Feret’s diameter after both treatments: control group (*p* < 0.05) and experimental group (*p* < 0.001). It is well-established that the cell nuclei of the stratified squamous gingival epithelium enlarge during gingival inflammation [24,25], a finding that is in accordance with the results of this study.

Igic et al. [26] assessed gingival inflammation through cytomorphometric analysis and found that following SRP treatment, the nuclei of the stratified squamous epithelial cells were reduced in size. However, after adjunctive low-level laser therapy (anti-inflammatory property), the nuclear size was reduced and comparable to that of epithelial cells in healthy individuals. These findings indicate that inflammation may lead to an enlargement of the nuclear area, but typically in young cells [27].

After treatment, the experimental group demonstrated significantly reduced values for nuclear area, perimeter, integrated optical density, and Feret’s diameter in comparison to the SRP control group, which suggests a notable decrease in nuclear and cellular activity linked to inflammation. These findings suggest that the adjunctive therapy effectively reduced inflammatory and reparative cellular responses, resulting in the restoration of epithelial homeostasis. The observed increase in circularity and nuclear roundness further supports this interpretation, as normalisation of nuclear morphology typically accompanies resolution of inflammatory processes and tissue regeneration. Similar reductions in nuclear dimensions after periodontal therapy have been reported in previous cytomorphometric studies, confirming that decreased nuclear size and increased regularity reflect diminished transcriptional activity and chromatin condensation during healing [3,5,27].

The selection of COX as a target for the subsequent molecular docking analysis, following clinical confirmation of the phytopreparation’s efficacy, is supported by previous studies showing that molecules selectively inhibiting COX-2 may serve as potential adjuncts in the treatment of periodontal disease [28]. This is further supported by the fact that COX-2 is a major mediator of inflammation in periodontitis, driving the loss of gingival tissue and the alveolar bone supporting the teeth [29,30]. Mechanistically, COX-2 catalyzes the conversion of arachidonic acid to prostaglandin H2 (PGH2). PGH2 is then converted to prostaglandin E2 (PGE2), which is released by gingival fibroblasts and other cell types and contributes to the pathogenesis of periodontitis [7].

Interpreting the data first by MolDock and then by Rerank, the clinically most meaningful narrative is the Rerank-based one. In this context, “clinically meaningful” refers to structure–compatibility and pose stability rather than to experimentally confirmed enzyme inhibition. The reason is mechanistic: the tincture contains both compact aglycones/phenolic acids and bulky glycosides. Across Table 4 and Table 5, the sign of the VdW term separates the isoforms much more sharply than raw MolDock totals or H-bond counts. In COX-1, the large glycosides encounter repulsive VdW contributions (e.g., rutin +40.034 kcal/mol; vitexin +84.202 kcal/mol), which pushes down their reranked affinity despite decent steric/H-bond terms; in COX-2, the same ligands see negative VdW values (rutin −41.118 kcal/mol; narcissoside −42.024 kcal/mol), aligning with a roomier pocket and yielding stable high Rerank Scores. By contrast, hyperoside, chlorogenic acid, rosmarinic acid, and to a lesser extent, isorhamnetin maintain negative VdW terms and strong steric complements in COX-1, explaining their higher COX-1 Rerank Scores even when their absolute H-bonding is similar to what they achieve in COX-2. Taken together, the Rerank analysis supports a complementary dual-target profile of the tincture, which is consistent with, but does not by itself demonstrate, functional COX inhibition in vivo. Narcissoside and rutin are the principal COX-2 binders, while hyperoside (top COX-1), chlorogenic acid, luteolin-7-*O*-glucoside, and rosmarinic acid contribute predominantly to COX-1 modulation. Luteolin-7-*O*-glucoside—with near-balanced reranked affinities—acts as a bridge across isoforms and may contribute to the overall interaction complementarity of the mixture. Accordingly, these docking results should be interpreted as a hypothesis-generating framework that rationalizes isoform compatibility, rather than as direct evidence of sustained inhibitory activity. The strong H-bonding observed for rosmarinic and chlorogenic acids explains their good absolute scores, yet it is the favorable VdW fit that ultimately governs isoform selectivity; where VdW becomes positive, the clinical plausibility of sustained binding decreases even if MolDock remains attractive.

This docking-based partitioning aligns well with the observed clinical regression of gingival inflammation after topical application. In inflamed gingiva, both isoforms contribute to local prostaglandin biosynthesis, and a mixture that simultaneously attenuates COX-1 and COX-2 would be expected to suppress PGE_2_ more effectively than any single constituent. The dataset also explains why a single dominant “hero” molecule is unlikely: the small phenolics (e.g., gallic acid) display excellent per-atom efficiency but limited total interaction energy, whereas the bulkier glycosides excel only in the isoform that can accommodate them without repulsion. The tincture’s net anti-inflammatory action therefore emerges most plausibly from functional synergy, wherein COX-2 is chiefly targeted by narcissoside/rutin, COX-1 by hyperoside/chlorogenic/rosmarinic, and luteolin-7-*O*-glucoside provides cross-isoform coverage. The Rerank-based analysis, with particular emphasis on the van der Waals energy component, allows discrimination between ligands with favorable volumetric complementarity and those subject to steric penalties within the cyclooxygenase channel. Compact phenolic acids and aglycones exhibit interaction patterns compatible with the narrower COX-1 active site, whereas bulkier flavonoid glycosides are more readily accommodated within the larger COX-2 pocket. These observations should be interpreted as structure–compatibility trends rather than quantitative predictions of inhibitory potency, and they primarily serve to rationalize how a chemically heterogeneous polyphenolic mixture may engage both isoforms in a complementary manner.

The 2D interaction maps for COX-1 (Figures S1–S9) and the docking readouts (MolDock Score and Rerank Score with per-term Steric, van der Waals, and HBond components, all in kcal/mol) converge on a coherent structure–activity picture. The active site exhibits a characteristic entry “gate” formed by Arg120 and Tyr355, a hydrophobic/*π*–*π* corridor that leads toward the catalytic region around Phe381, Tyr385, and Trp387, and a polar neighborhood near Ser530. Poses that simultaneously engage this triad—firm anchoring at the Arg120/Tyr355 gate, contiguous hydrophobic/*π*–*π* packing along the channel, and occasional stabilization near Ser530—yield strong Steric contributions and attractive MolDock Scores. After reranking, they retain favorable Rerank values so long as the Lennard-Jones van der Waals (VdW) term remains negative, indicating good volumetric complementarity without clashes.

Across classes of secondary metabolites, the maps rationalize why MolDock and Rerank sometimes diverge and which metric is more clinically informative for COX-1. Phenolic acids (e.g., chlorogenic, rosmarinic, gallic) consistently show bidentate or multidentate anchoring at the gate, with dense hydrogen bonding and a continuous strip of hydrophobic contacts down the channel, resulting in strongly favorable HBond contributions and persistently negative VdW; their attractive MolDock Scores therefore survive the reranking step and often improve. Luteolin-7-*O*-glucoside and isorhamnetin display compact aromatic packing and *π*–*π*/cation–*π* contacts through the core of the pocket, typically supported by one or two strategic hydrogen bonds (often the flavone carbonyl as an acceptor). This “clean” interaction topology produces robust Steric terms, negative VdW, and good agreement between MolDock and Rerank, explaining why these aglycones frequently behave as isoform “bridges” in the broader dataset.

In contrast, flavonoid glycosides (e.g., rutin, narcissoside, vitexin) produce 2D maps rich in hydrogen bonds owing to saccharide donors/acceptors. Yet, their saccharide moieties often spill across the channel entrance and crowd the Arg120/Tyr355 region. MolDock—driven by overall shape complementarity and HBonding—can still appear attractive for such bulky ligands if the aromatic core packs reasonably well; however, the Rerank Score, which reweights VdW and penalizes steric clashes, unmasks the practical cost of entrance congestion: the VdW term becomes less favorable or even positive, and Rerank deteriorates despite a decent MolDock. Notable exceptions occur when the glycoside adopts a tidier pose (e.g., hyperoside in specific orientations), in which 2D maps show well-distributed contacts: sugars and the aglycone form hydrogen bonds without visible crowding at the gate. At the same time, the aromatic scaffold maintains a continuous hydrophobic track. In these cases, VdW remains negative, and the Rerank Score aligns with MolDock, indicating the pose is genuinely compatible with the COX-1 pocket.

Overall, the combined evidence from the maps and the score decompositions indicates that Rerank more faithfully captures pocket compatibility in COX-1 because it encodes the volumetric penalties that the 2D maps make visually apparent. Compact phenolic acids and aglycones—those that engage the Arg120/Tyr355 gate and sustain uninterrupted packing—emerge as the more reliable COX-1 binders after reranking. In contrast, the largest glycosides underperform whenever saccharide crowding induces VdW penalties. This structure–score correspondence also helps contextualize the isoform patterns shown in the tables: ligands penalized in COX-1 by entrance congestion tend to fare better in the roomier COX-2 pocket (where VdW interactions become favorable). At the same time, compact scaffolds retain strong COX-1 engagement. In practical terms, this explains why a polyphenolic tincture can act through complementary contributions: compact phenolics provide robust COX-1 coverage, bulky glycosides express their full potential in COX-2, and balanced aglycones (e.g., luteolin-7-*O*-glucoside) bridge the two, together yielding the multi-target inhibition profile that matches the observed regression of gingival inflammation.

The 2D interaction maps for COX-2 (Figures S10–S18) together with the docking readouts (MolDock Score and Rerank Score with Steric, van der Waals, and HBond components, all in kcal/mol) point to a binding regime that is visibly more permissive to bulky, hydrogen-bond-rich scaffolds than COX-1. In COX-2, ligands still exploit the canonical “gate” defined by Arg120 and Tyr355, traverse a hydrophobic/*π*–*π* corridor toward the catalytic region around Phe381, Tyr385, and Trp387, and engage polar functionality near Ser530; however, the enlarged channel and accessible side pocket in COX-2 allow voluminous substituents—especially saccharide moieties of flavonoid glycosides—to be accommodated without the entrance crowding that penalizes them in COX-1. On the 2D maps, this appears as dense hydrogen-bond networks at (or just past) the gate, coupled with extended hydrophobic contacts that continue deeper into the pocket, often spilling into the lateral subcavity; in the scoring decomposition this translates to strongly favorable HBond terms and, crucially, negative van der Waals (VdW) contributions after reranking, a signature of good volumetric complementarity rather than steric clash.

Phenolic acids (e.g., chlorogenic and rosmarinic) anchor reliably at Arg120/Tyr355 via multiple hydrogen bonds and form a continuous strip of hydrophobic contacts along the channel. Their per-term pattern is therefore predictable: attractive HBond, consistently negative VdW, and solid Steric packing, yielding MolDock Scores that remain robust—or modestly improve—upon Rerank. Small acids such as gallic display high per-atom efficiency but a limited absolute interaction surface; on the maps, this is evident as shorter hydrophobic tracks, which explains why their total scores sit below those of larger polyphenols despite excellent hydrogen-bonding geometry.

Luteolin-7-*O*-glucoside and isorhamnetin present “clean” COX-2 interaction topologies: compact aromatic scaffolds pack deeply and continuously through the channel core, establishing *π*–*π* (and occasional cation–*π*) contacts and one or two stabilizing hydrogen bonds, often via the flavone carbonyl as an acceptor. Because the aglycones do not protrude excessively into the gate region, their VdW terms remain negative and closely track their Steric contributions; as a result, MolDock and Rerank are in good agreement and mark these ligands as reliable, isoform-bridging binders. By contrast, the flavonoid glycosides (e.g., rutin, narcissoside, hyperoside, vitexin) make profuse hydrogen bonds through their sugar residues and, in COX-2, can place those residues without congesting the gate. The 2D maps typically show a tidy distribution: the saccharide establishes multiple H-bonds at or near the entrance. At the same time, the aromatic core extends a contiguous hydrophobic contact strip into the deeper cavity and side pocket. This geometry is precisely what the scoring terms reward in COX-2: HBond remains strongly favorable and, unlike in COX-1, the VdW contribution stays negative rather than turning positive, so the Rerank Score corroborates (rather than downgrades) the initially attractive MolDock pose. Occasional poses that underperform after reranking can usually be traced, on the maps, to misoriented sugars that partially occlude the entrance; even then, the penalty is milder than in COX-1 because the COX-2 cavity provides additional space for relief.

Collectively, these observations establish a consistent correspondence between structure and score for COX-2. The features that the 2D maps make visually explicit—robust gate anchoring, uninterrupted hydrophobic/*π*–*π* packing, and spatial relief for bulky substituents—are mirrored numerically by attractive Steric terms, strong HBond energies, and negative VdW contributions in the reranked poses. Consequently, glycosides emerge as powerful COX-2 binders once Rerank is considered, while phenolic acids and compact aglycones remain dependable contributors with slightly lower absolute scores but excellent efficiency and pose stability. Mechanistically, this explains why the polyphenol mixture derived from the tincture can deliver potent COX-2 engagement without sacrificing binding quality: large, H-bond–rich ligands exploit COX-2’s extra volume rather than being penalized by it, and smaller scaffolds fill remaining space efficiently. This complementarity aligns with the clinical observation of inflammation regression. It dovetails with the COX-1 analysis to support a multi-target, synergistic mechanism in which glycosides primarily drive COX-2 inhibition, while acids and aglycones contribute broad-spectrum stability across both isoforms.

Accordingly, the proposed mechanistic interpretation should be regarded as a rational, hypothesis-generating framework linking chemical composition with biological plausibility. Definitive confirmation of COX-mediated effects will require targeted biochemical assays or ex vivo inflammatory readouts in future studies.

The concept of in silico evaluation of drug-likeness and ADME/Tox properties of phytocompounds for periodontitis treatment reflects a contemporary approach [31,32], which has also been applied to other oral diseases [33,34]. It has emerged due to the high cost, prolonged development time, and elevated attrition rates associated with conventional drug discovery, aiming to prioritize compounds with favorable ADME/Tox properties [35].

Drug-likeness is most commonly assessed according to Lipinski’s Rule of Five (Ro5), which predicts oral bioavailability and membrane permeability of molecules based on their physicochemical properties [36]. According to Ro5, a compound is expected to have high gastrointestinal absorption if its molecular weight is ≤500 g/mol, log Po/w is ≤5, the number of hydrogen bond acceptors is ≤10, the number of hydrogen bond donors is ≤5, and the number of rotatable bonds is ≤10. Additionally, experimental evidence suggests that compounds should have a topological polar surface area (TPSA) ≤ 140 Å^2^ [36]. Although violations of these rules may indicate potential pharmacokinetic challenges, they do not necessarily preclude drug development, particularly for topical applications.

In the current study, the predicted low gastrointestinal absorption and inability to cross the BBB indicate limited systemic exposure of the majority of tested phenolic compounds. The predicted high gastrointestinal absorption of gallic acid and isorhamnetin may be attributed to their relatively low molecular weight, which generally favours membrane permeation and absorption [35]. Low gastrointestinal absorption, together with highly negative skin permeation values, suggests their local retention at the site of application, which may be advantageous for topical use in the gingival tissue. Moreover, the predicted lack of P-gp substrate activity and weak CYP inhibition further reduces the risk of systemic drug–drug interactions. Overall, the tested molecules exhibited generally low predicted toxicity profiles for topical application. Indeed, although the in silico analysis indicated “active” signals for certain toxicity endpoints (Table 8), including predicted nephrotoxicity, respiratory toxicity, and hERG II inhibition for some compounds, the relevance of these predicted risks should be interpreted in the context of local, short-term, and low-dose gingival application, where limited absorption and minimal systemic exposure are expected. Accordingly, these signals warrant cautious interpretation and further validation, rather than being viewed as definitive safety concerns.

Combined with the absence of side effects observed during the 1-month clinical study, these in silico results support the potential of the tested phytopreparation and its phenolic constituents as safe and locally acting agents for managing periodontal disorders. Nevertheless, although no local or systemic adverse reactions were observed during this short-term study, the results do not exclude potential risks associated with long-term or improper use, or the occurrence of rare adverse effects that may only be detectable in larger populations. Combined with generally low toxicity profiles, these in silico results support the potential of these phenolic compounds as safe and locally acting agents for managing periodontal disorders. Although advanced in silico tools offer considerable promise for ADME/Tox evaluation, their reliance on datasets of synthetic compounds may limit applicability to natural products. Therefore, validation through in vivo and clinical studies remains essential [35,37]. The cytomorphometric and in silico findings should be viewed as exploratory but biologically supportive, providing complementary mechanistic evidence for the anti-inflammatory and tissue-modulating effects of the phytotherapeutic formulation.

## 4. Materials and Methods

### 4.1. Study Design

**Figure 2:**
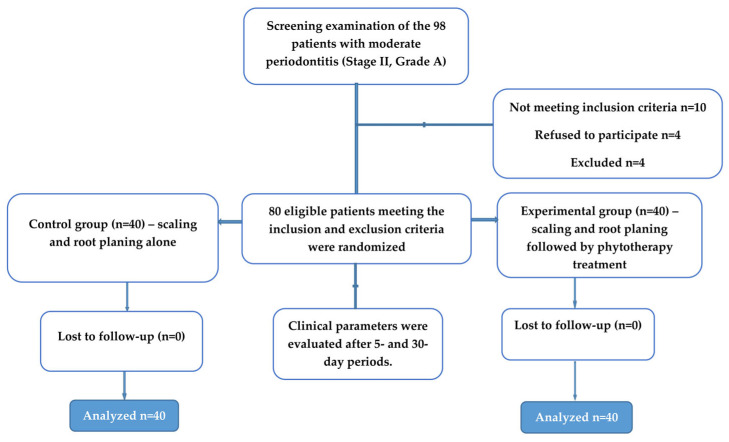
Study outline.

The protocol for preparation, administration, and clinical use of the polyherbal phytopreparation (Tinctura paradentoica^®^, Institute “Dr Josif Pančić,” Belgrade, Serbia) followed our previously published clinical study [15]. Briefly, 80 patients with moderate periodontitis (Stage II, Grade A) were randomised to receive either scaling and root planing therapy (SRP) alone or SRP followed by phytotherapeutic treatment. Control (SRP) group—40 patients with periodontitis who received SRP treatment by ultrasonic scaler Woodpecker (UDS-J, Medical Instrument Company, Guilin, Guangxi, China) and curettage with curettage of periodontal pockets (with Gracey 5–6, hand curettes Hu Friedy, Chicago, Il, USA) for five consecutive days. The experimental group—40 patients with periodontitis who received local phytotherapy after causal periodontal therapy. All patients received oral hygiene instructions (Figure 2).

Randomisation was computer-generated with allocation concealment, and both participants and the recruiting investigator were blinded to group assignment. Group assignment was carried out using a computer-based randomization method with equal block sizes to achieve comparable distribution between the study arms. To preserve allocation concealment, sequentially numbered, opaque, sealed envelopes were prepared by an independent examiner who had no involvement in participant enrollment or outcome evaluation. Blinding was ensured, as both the participants and the investigators responsible for recruitment were unaware of treatment allocation at the time of inclusion. All clinical assessments were conducted by a single, calibrated examiner who remained blinded to the assigned interventions and who was not involved in treatment delivery. Microbiological analyses were performed by laboratory personnel using coded samples, and the analysts had no knowledge of the participants’ group assignments.

The treatment consisted of polishing, ultrasonic scaling, root planing, and pocket curettage over five consecutive days by a calibrated periodontist. In the intervention group, 0.1 mL of tincture was applied subgingivally per pocket using a sterile syringe with a 23-gauge needle (110° angulation) for two minutes per quadrant under cotton roll isolation. The composition of the polyherbal phytopreparation under investigation is shown in Table 9.

### 4.2. Ethical Approval

The research was approved by the Faculty of Medicine, University of Niš, Republic of Serbia (Approval No. 12-6422-2/7), and written informed consent was obtained from all participants prior to their inclusion in the study. This study was registered retrospectively with the clinical trial registration number ISRCTN10262904 (https://www.isrctn.com/ISRCTN10262904, accessed on 20 February 2026).

### 4.3. Inclusion Criteria

Participants suffering from periodontitis were selected following a medical history assessment and a clinical evaluation. The selection criteria included systemically healthy individuals with a natural dentition of at least 24 teeth and radiographic confirmation of bone loss. Periodontal status was determined based on the 2018 classification of periodontal diseases and conditions [38]. A diagnosis of periodontitis was assigned if the patient exhibited interdental clinical attachment loss (CAL) at two or more non-adjacent teeth, or buccal/oral CAL ≥ 3 mm in conjunction with periodontal pocket depth (PPD) ≥ 3 mm at two or more teeth. Participants who did not fulfill these criteria were categorized as periodontally healthy.

### 4.4. Exclusion Criteria

Participants were not included in the study if they had a history of alcohol consumption; tobacco use in any form (past or present); or a medical history of anemia, diabetes, hepatitis, tuberculosis, AIDS, leukemia, or other systemic or hormonal disorders linked to gingival manifestations. Additionally, those who were undergoing or had previously undergone treatment with systemic hormonal therapy, contraceptive usage, corticosteroids, immunosuppressants, radiation therapy, or chemotherapy before the study started were also excluded.

### 4.5. Clinical Evaluation

Before the study began, the examiner completed calibration training with a two-day interval. Calibration and measurement consistency were verified by duplicating assessments of five periodontitis patients who were not part of the study, with 96% of repeated measurements differing by no more than 1 mm. All periodontal measurements were conducted by a single periodontist using a manually calibrated periodontal probe (PQWBR—Hu Friedy Mfg. Inc., Chicago, IL, USA).

Before and after treatment, oral hygiene levels were assessed, and gingival and periodontal measurements were taken using specific indices: The Löe–Silness Gingival Index (GI) was used to assess the condition of the gingiva, excluding third molars and retained root fragments. Measurements were taken on the axial surfaces of the teeth, including vestibular, oral, and proximal sides. The overall gingival index was calculated by summing the index values from all tooth surfaces and dividing the total by four. The clinical condition of the gingiva was classified using a numerical scale ranging from 0 to 3. A score of 0.1–1.0 indicates mild inflammation, 1.1–2.0 represents moderate inflammation, and 2.1–3.0 signifies severe inflammation. Periodontal pocket depth (PPD) values were recorded in millimeters. Measurements were taken at six distinct sites per tooth—mesio-buccal, mid-buccal, disto-buccal, mesio-lingual, mid-lingual, and disto-lingual—rounded to the nearest whole millimeter.

### 4.6. Cytomorphometric Analysis

Swabs from the gingiva were taken from all participants before therapy, and during the control examination, a month after therapy. The sample was applied to a clean, non-greased microscope slide and uniformly spread as a thin layer. It was air-dried at ambient temperature (for at least one hour), fixed in 96% ethanol for 10 min, and subsequently stained with the Papanicolaou method [30]. Following staining, the sections of the slides were mounted with coverslips and sealed using DPX (dibutyl phthalate xylene) glue medium. The rapid Papanicolaou staining, CYTOSEMICOLOR (Semikem d.o.o., Sarajevo, Bosnia and Herzegovina), was performed according to the following procedure: reagents were added to staining trays in the prescribed order. After fixation with Sprayfix^®^ spray, (Leica Biosystems, Deer Park, IL, USA) the specimen was immersed in the following solutions: distilled water 10 × 1 s, Harris’s modified hematoxylin 1 × 1 min, then rinse under a water jet 1 × 5 s, 2-propanol 2 × 1 s, polychromatic modified solution 1 × 1 min, 2-propanol 80% 5 × 1 s, 2-propanol 5 × 1 s, 2-propanol 5 × 1 s, xylene 5 × 1 s and xylene 5 × 1 s [39].

The nuclear area (μm^2^), perimeter (μm), integrated optical density (IntDen), nuclear roundness (round), circularity (circ), and Feret’s diameter (μm) were measured in cytological samples using ImageJ software (https://imagej.net/ij/download.html, accessed on 1 December 2024, version 1.53k. Public domain software. Wayne Rasband, National Institutes of Health, Bethesda, MD, USA). The analysis was performed with an Olympus BX50 (Carl Zeiss, Tokyo, Japan) optical microscope at 40× magnification (numerical aperture 0.75). Epithelial cell nuclei were manually selected with a computer mouse.

### 4.7. Molecular Docking Analysis

Molecular docking was performed in Molegro Virtual Docker (MVD, v6.0) on the HPLC-identified constituents against cyclooxygenase isoforms COX-1 and COX-2 [40]. High-resolution crystallographic structures of the two isoforms (with co-crystallized inhibitors) were retrieved from the Protein Data Bank (pdb: 6y3c and 3ln) and preprocessed by removing nonessential waters, adding polar hydrogens, and assigning protonation states appropriate for physiological pH. The binding region was centered to encompass the canonical cyclooxygenase channel—including the Arg120/Tyr355 “gate,” the hydrophobic/*π*–*π* corridor toward Tyr385 and the vicinity of Ser530, and extended to cover the adjacent side pocket in COX-2. Ligand geometries were energy-minimized prior to docking, multiple poses were generated per compound, and the top pose for each ligand was selected using MolDock Score and Rerank Score. For each ligand–protein complex, a series of energy terms and scores was calculated and reported in kcal/mol to characterize binding affinity and interaction quality. The MolDock Score served as the primary docking metric, integrating a piecewise linear potential (PLP) to model steric interactions, a directional term for hydrogen bonding, and an intramolecular energy contribution for ligand flexibility. More negative MolDock Scores indicated stronger predicted binding. To refine pose ranking and better account for pocket compatibility, the Rerank Score was applied as a post-processing step. This score re-weighted the initial MolDock contributions, placing greater emphasis on steric fit, van der Waals complementarity, and hydrogen-bond geometry while more heavily penalizing clashes and suboptimal volumetric filling. As a result, the Rerank Score generally provided a more reliable indicator of biologically relevant binding than the raw MolDock Score. Individual energy components were decomposed to enable detailed structure–activity analysis. The Steric term, based on a piecewise linear potential, captured short-range repulsive and attractive forces arising from atomic overlaps and ideal contact distances. The van der Waals (VdW) term, modeled using a Lennard-Jones 12-6 potential, accounted for medium-range dispersion and repulsion; negative values indicated favorable packing, whereas positive values indicated net repulsion due to steric clashes. The HBond term quantified the energetic contribution of hydrogen bonds, with more negative values corresponding to stronger and/or more numerous directional interactions between ligand and protein residues. The Energy term represented the total interaction energy of the pose, encompassing all steric, van der Waals, hydrogen-bonding, and intramolecular components. Ligand efficiency was assessed using two normalized indices. Ligand efficiency 1 (LE1) was calculated as the MolDock Score divided by the number of heavy atoms in the ligand, providing a measure of binding affinity per heavy atom. Similarly, ligand efficiency 3 (LE3) was defined as the Rerank Score divided by the number of heavy atoms, offering a rerank-adjusted efficiency metric. A rarely invoked penalty, NoHBond90, was included to account for hydrogen bonds deviating by more than 90° from ideal geometry, though it contributed minimally in most cases.

### 4.8. In Silico ADME/Tox Profiling

Computational analyses were performed to predict the physicochemical, drug-likeness, pharmacokinetic, and toxicity profiles of nine phenolic compounds (three phenolic acids and six flavonoids) previously quantified in Tinctura paradentoica*^®^* by HPLC-DAD [13]. The chemical structures were obtained from the PubChem (https://pubchem.ncbi.nlm.nih.gov/) database as canonical SMILES strings, and analyses were carried out using SwissADME (http://www.swissadme.ch/), ProTox-3.0 (https://tox.charite.de/protox3/), and pkCSM (https://biosig.lab.uq.edu.au/pkcsm/prediction) (all accessed on 10 September 2025).

Drug-likeness was evaluated using SwissADME by applying the Lipinski, Ghose, Veber, Egan, and Muegge filters, based on physicochemical parameters including molecular weight, number of rotatable bonds, number of hydrogen bond acceptors and donors, topological polar surface area (TPSA), and lipophilicity (log Po/w). Pharmacokinetic parameters (ADME parameters) predicted with SwissADME included gastrointestinal absorption, skin permeation, blood–brain barrier (BBB) penetration, interaction with permeability glycoprotein (P-gp), and potential metabolism by cytochrome P450 (CYP) isoenzymes (CYP1A2, CYP2C19, CYP2C9, CYP2D6, and CYP3A4).

Toxicity prediction was carried out using ProTox-3.0, which provided estimates of toxicity class based on LD_50_ values, organ-specific toxicities (hepatotoxicity, neurotoxicity, nephrotoxicity, respiratory toxicity, and cardiotoxicity), and toxicity endpoints including carcinogenicity, immunotoxicity, mutagenicity, cytotoxicity, BBB-related toxicity, ecotoxicity, clinical toxicity, and nutritional toxicity. Additionally, pkCSM was used to predict AMES mutagenicity, skin sensitization, and potential hERG I and hERG II inhibition.

### 4.9. Statistical Analysis

Statistical analyses were performed using SPSS software, version 15.0 (IBM Corp., USA). Continuous variables were summarized as mean values (X), standard deviations (SD), and medians (Me), whereas categorical variables were reported as absolute frequencies and corresponding percentages. Intergroup comparisons of categorical variables were conducted using Pearson’s chi-square test with the Mantel-Haenszel correction. The Shapiro–Wilk test was employed to evaluate the normality of distribution for continuous variables. Depending on the distributional characteristics, comparisons of independent samples were carried out using either Student’s *t*-test or the Mann–Whitney U test. In contrast, comparisons of paired samples utilised either the paired Student’s *t*-test or the Wilcoxon signed-rank test. Statistical significance was established at *p* < 0.05.

For between-group comparisons based on the Mann–Whitney U test, effect sizes were estimated using rank-biserial correlation (r_rb_), with 95% confidence intervals.

The primary clinical endpoint was the change in the gingival index (GI) from baseline to one month, reflecting gingival inflammation as the main therapeutic target. Periodontal pocket depth (PPD) was evaluated as a key secondary clinical outcome. Cytomorphometric variables were included as exploratory biological measures, while in silico docking and pharmacokinetic analyses were performed to provide mechanistic support rather than for confirmatory hypothesis testing.

To account for multiple testing across the primary and secondary clinical endpoints, *p*-values for between-group comparisons of GI and PPD were adjusted using the Holm–Bonferroni method. The cytomorphometric and in silico outcomes were analyzed in an exploratory framework and interpreted accordingly.

## 5. Conclusions

In summary, the results indicate that causal periodontal treatment, particularly when combined with a topical polyherbal phytopreparation, has effectively reduced both clinical and cytomorphometric parameters. This approach has proven to be more effective in controlling the inflammatory process during the experimental period. The insights gained from these findings lay the groundwork for future phytotherapeutic approaches aimed at enhancing causal periodontal therapy. The accompanying docking study, guided by HPLC-identified constituents, does not constitute direct evidence of enzymatic inhibition but supports a biologically plausible, multi-target anti-inflammatory framework. Docking results suggest complementary compatibility of structurally diverse phenolic compounds with COX-1 and COX-2, with compact phenolic acids and aglycones favoring COX-1 and bulkier glycosides showing preferential accommodation within the COX-2 pocket. Together, these findings provide a coherent translational rationale for the observed clinical benefits, while emphasizing the need for future biochemical or ex vivo studies to validate the proposed molecular mechanisms. The in silico ADME/Tox profiling of the phenolic compounds from the investigated phytopreparation suggests generally favourable pharmacokinetic and safety characteristics, including low systemic absorption, with no predicted mutagenicity or skin sensitisation potential. These results highlight their potential for safe topical application in the treatment of periodontitis. Further long-term clinical studies are required to verify the observed efficacy and safety potential of the investigated phytopreparation. This study was conducted as an academic clinical investigation in accordance with ethical principles and Good Clinical Practice at the Clinic of Dentistry, Faculty of Medicine, University of Niš, Serbia. It was not designed as a regulatory clinical trial for drug authorization under the FDA or EMA frameworks or other healthcare organizations.

## Figures and Tables

**Table 1:** Demographic characteristics of study participants (results are represented as mean with standard deviation in parentheses).

Characteristics	Control Group (*n* = 40)	Experimental Group (*n* = 40)
Age (years)	42.63 (13.95)	44.83 (14.79)
Gender (%)		
Male	15 (37.5%)	15 (37.5%)
Female	25 (62.5%)	25 (62.5%)

**Table 2:** Gingival index (GI), before, after five days and a month after therapy, Periodontal Pocket Depth (PPD) before and a month after therapy (results are presented as the mean with standard deviation in parentheses).

Index	Control (SRP) Group		Experimental Group	
GI Before	1.30	(0.51) ^$$$,^***	1.39	(0.50) ^$$$,^***
After five days	0.52	(0.49) ^##^	0.22	(0.45)
After a month	0.66	(0.69) ^#^	0.44	(0.51)
PPD Before	4.75	(0.27) ***	4.72	(0.29) ***
After a month	4.17	(0.44) ^###^	4.09	(0.39)

^$^—vs. after the fifth therapy, *—vs. a month after therapy (Wilcoxon Signed Ranks Test). ^#^—vs. experimental group (Mann–Whitney test). ^#^ *p* < 0.05; ^##^ *p* < 0.01; ^$$$^/***/^###^ *p* < 0.001.

**Table 3:** Results of cytomorphometric analysis before and a month after therapy (results are presented as the mean with standard deviation in parentheses).

Parameters	Control (SRP) Group		Experimental Group	
Nuclear area (μm^2^)				
Before	61.17	(13.51) *	60.82	(12.59) ***
After	56.98	(16.68) ^###^	39.43	(9.11)
Perimeter (μm)				
Before	30.20	(3.54) *	29.05	(3.11) ***
After	29.03	(3.94) ^###^	23.14	(2.70)
Ferret’s diameter (μm)				
Before	10.51	(1.16) *	10.51	(1.14) ***
After	10.07	(1.36) ^###^	8.30	(0.98)
Integrated optical density (IntDen)				
Before	23.32	(5.03) *	26.68	(5.29) ***
After	21.40	(5.58) ^###^	17.86	(0.98)
Nuclear roundness (round)				
Before	0.75	(0.08)	0.75	(0.10) **
After	0.76	(0.09) ^##^	0.80	(0.09)
Circularity (circ)				
Before	0.83	(0.07)	0.89	(0.06) *
After	0.83	(0.07) ^###^	0.91	(0.05)

^#^—statistically significant difference vs. experimental group (Mann–Whitney test, Student’s *t* test for independent samples), *—statistically significant difference vs. a month after therapy (Wilcoxon Signed Ranks Test, Paired samples *t*-tests). * *p* < 0.05; **/^##^ *p* < 0.01; ***/^###^ *p* < 0.001.

**Table 4:** Docking results for HPLC-identified tincture constituents against COX-1: MolDock Score, Rerank Score, and per-term energy components (van der Waals and hydrogen-bond), all in kcal/mol.

Molecule	Energy	HBond	Ligand Efficiency 1 (LE1)	Ligand Efficiency 3 (LE3)	NoHBond90	MolDock Score	Rerank Score	Steric	VdW
Rosmarinic acid	−168.08	−29.293	−6.465	−5.069	−34.999	−190.606	−131.799	−154.694	−33.924
Hyperoside	−160.122	−28.122	−4.852	−4.428	−32.327	−210.204	−146.114	−190.964	−40.155
Luteolin-7-*O*-glucoside	−159.122	−19.700	−4.972	−4.227	−23.661	−193.250	−135.262	−176.374	−37.292
Rutin	−148.323	−22.684	−3.449	−2.031	−29.542	−197.442	−87.331	−179.286	40.034
Narcissoside	−146.514	−22.628	−3.329	−2.375	−33.034	−200.646	−104.501	−144.496	−28.360
Vitexin	−145.889	−22.022	−4.706	−1.981	−26.875	−175.603	−61.421	−158.865	84.201
Chlorogenic acid	−138.887	−20.431	−5.555	−5.419	−23.945	−160.898	−135.500	−157.655	−56.202
Isorhamnetin	−121.185	−17.970	−5.269	−4.715	−21.719	−137.246	−108.449	−137.634	−36.308
Gallic acid	−87.832	−21.130	−7.319	−6.424	−23.578	−94.878	−77.089	−75.024	−25.458

**Table 5:** Docking results for HPLC-identified tincture constituents against COX-2: MolDock Score, Rerank Score, and per-term energy components (van der Waals and hydrogen-bond), all in kcal/mol.

Molecule	Energy	HBond	Ligand Efficiency 1 (LE1)	Ligand Efficiency 3 (LE3)	NoHBond90	MolDock Score	Rerank Score	Steric	VdW
Rosmarinic acid	−167.529	−17.056	−6.443	−4.472	−20.036	−168.768	−116.269	−124.951	−6.587
Luteolin-7-*O*-glucoside	−165.160	−11.399	−5.161	−4.126	−14.904	−199.463	−132.053	−117.733	−38.062
Narcissoside	−156.528	−19.567	−3.557	−3.153	−30.770	−212.057	−138.733	−180.825	−42.024
Rutin	−155.648	−19.973	−3.619	−3.186	−30.707	−210.146	−137.017	−177.953	−41.118
Hyperoside	−137.133	−12.885	−4.155	−3.392	−12.885	−180.793	−111.951	−94.855	−30.346
Chlorogenic acid	−133.290	−24.802	−5.331	−4.725	−29.169	−161.576	−118.126	−128.721	−30.343
Vitexin	−122.136	−13.152	−3.939	−1.817	−17.348	−147.735	−56.3463	−81.909	51.828
Isorhamnetin	−121.036	−11.172	−5.262	−4.143	−11.935	−128.252	−95.2926	−118.350	−19.315
Gallic acid	−86.209	−7.496	−7.184	−5.089	−7.496	−93.636	−61.0791	−27.563	−7.367

**Table 6:** In silico assessment of physicochemical and drug-likeness properties of phenolic compounds from Tinctura paradentoica*^®^* using SwissADME software (http://www.swissadme.ch, accessed on 10 September 2025).

Molecule	Phenolic Acids			Flavonoids					
	Chlorogenic Acid	Gallic Acid	Rosmarinic Acid	Rutin	Isorhamnetin	Narcissoside	Vitexin	Luteolin-7-*O*-glucoside	Hyperoside
PubChem CID	1794427	370	5281792	5280805	5281654	5481663	5280441	45933934	5281643
Molecular weight (g/mol)	354.31	170.12	360.31	360.31	316.26	624.54	432.38	448.38	464.38
Number of rotatable bonds	5	1	7	7	2	7	3	4	4
Number of H-bond acceptors	9	5	8	8	7	16	10	11	12
Number of H-bond donors	6	4	5	5	4	9	7	7	8
TPSA (Å^2^)	164.75	97.99	144.52	144.52	120.36	258.43	181.05	190.28	210.51
Lipophilicity (Consensus Log Po/w)	−0.39	0.21	1.58	1.58	1.65	−0.8	−0.02	0.19	−0.38
Water solubility (Log S (Ali))	−2.58	−2.34	−5.04	−5.04	−4.02	−4.97	−3.57	−5.06	−4.35
Solubility class (Ali class)	Soluble	Soluble	Moderately soluble	Moderately soluble	Moderately soluble	Moderately soluble	Soluble	Moderately soluble	Moderately soluble
Lipinski	Yes *	Yes	Yes	Yes	Yes	No ***	Yes *	No **	No **
Ghose	No *	No **	Yes	Yes	Yes	No ****	Yes	Yes	No *
Veber	No *	Yes	No *	No *	Yes	No *	No *	No **	No *
Egan	No *	Yes	No *	No *	Yes	No *	No *	No *	No *
Muegge	No **	No *	Yes	Yes	Yes	No ****	No **	No ***	No ***
Bioavailability Score	0.11	0.56	0.56	0.56	0.55	0.17	0.55	0.17	0.17

TPSA—topological polar surface area; *—1 violation; **—2 violations; ***—3 violations; ****—4 violations.

**Table 7:** In silico assessment of pharmacokinetic properties of phenolic compounds from Tinctura paradentoica*^®^* using SwissADME software.

Molecule	Phenolic Acids			Flavonoids					
	Chlorogenic Acid	Gallic Acid	Rosmarinic Acid	Rutin	Isorhamnetin	Narcissoside	Vitexin	Luteolin-7-*O*-glucoside	Hyperoside
GIT absorption	Low	High	Low	Low	High	Low	Low	Low	Low
BBB permeant	No	No	No	No	No	No	No	No	No
P-gp substrate	No	No	No	No	No	Yes	No	Yes	No
CYP1A2 inhibitor	No	No	No	No	Yes	No	No	No	No
CYP2C19 inhibitor	No	No	No	No	No	No	No	No	No
CYP2C9 inhibitor	No	No	No	No	No	No	No	No	No
CYP2D6 inhibitor	No	No	No	No	Yes	No	No	No	No
CYP3A4 inhibitor	No	Yes	No	No	Yes	No	No	No	No
Skin permeation (log Kp (cm/s))	−8.76	−6.84	−6.82	−6.82	−6.9	−10.12	−8.79	−8	−8.88

GIT—gastrointestinal; BBB—blood–brain barrier; P-gp—permeability glycoprotein; CYP—cytochrome P450.

**Table 8:** In silico assessment of toxicity properties of phenolic compounds from Tinctura paradentoica*^®^* using ProTox-3.0 and pkCSM software (https://tox.charite.de/protox3/, https://bosig.lab.uq.edu.au/pkcsm/prediction, accessed on 10 September 2025).

Molecule	Phenolic Acids			Flavonoids					
	Chlorogenic Acid	Gallic Acid	Rosmarinic Acid	Rutin	Isorhamnetin	Narcissoside	Vitexin	Luteolin-7-*O*-glucoside	Hyperoside
LD_50_ (mg/kg)	5000	2000	5000	5000	5000	5000	832	5000	5000
Toxicity class	5	4	5	5	5	5	4	5	5
Hepatotoxicity *	Inactive (0.72)	Inactive (0.88)	Inactive (0.62)	Inactive (0.8)	Inactive (0.72)	Inactive (0.81)	Inactive (0.81)	Inactive (0.82)	Inactive (0.82)
Neurotoxicity *	Inactive (0.8)	Inactive (0.69)	Inactive (0.88)	Inactive (0.89)	Inactive (0.88)	Inactive (0.87)	Inactive (0.88)	Inactive (0.88)	Inactive (0.88)
Nephrotoxicity *	Active (0.5)	Active (0.52)	Active (0.64)	Active (0.77)	Active (0.64)	Active (0.76)	Active (0.67)	Active (0.76)	Active (0.76)
Respiratory toxicity *	Active (0.57)	Active (0.89)	Inactive (0.54)	Active (0.63)	Active (0.85)	Active (0.66)	Active (0.66)	Active (0.61)	Active (0.61)
Cardiotoxicity *	Inactive (0.99)	Inactive (0.56)	Inactive (0.69)	Inactive (0.98)	Active (0.82)	Inactive (0.53)	Inactive (0.63)	Inactive (0.67)	Inactive (0.67)
Carcinogenicity *	Inactive (0.68)	Active (0.99)	Inactive (0.66)	Inactive (0.91)	Inactive (0.68)	Inactive (0.93)	Inactive (0.72)	Inactive (0.85)	Inactive (0.85)
Immunotoxicity *	Active (0.99)	Inactive (0.94)	Active (0.93)	Active (0.98)	Active (0.58)	Active (0.99)	Inactive (0.82)	Inactive (0.74)	Active (0.66)
Mutagenicity *	Inactive (0.93)	Inactive (0.91)	Inactive (0.85)	Inactive (0.88)	Inactive (0.94)	Inactive (0.9)	Active (0.52)	Inactive (0.76)	Inactive (0.76)
Cytotoxicity *	Inactive (0.8)	Inactive (0.67)	Inactive (0.9)	Inactive (0.64)	Inactive (0.95)	Inactive (0.52)	Inactive (0.87)	Inactive (0.69)	Inactive (0.69)
BBB-barrier toxicity *	Active (0.6)	Active (0.8)	Active (0.62)	Inactive (0.75)	Active (0.52)	Inactive (0.99)	Inactive (0.55)	Inactive (0.57)	Inactive (0.57)
Ecotoxicity *	Inactive (0.72)	Inactive (0.55)	Inactive (0.79)	Inactive (0.6)	Inactive (0.55)	Inactive (0.58)	Inactive (0.59)	Inactive (0.58)	Inactive (0.58)
Clinical toxicity *	Active (0.67)	Active (0.83)	Active (0.59)	Active (0.52)	Inactive (0.5)	Active (0.53)	Active (0.51)	Active (0.51)	Active (0.51)
Nutritional toxicity *	Inactive (0.64)	Inactive (0.88)	Inactive (0.65)	Active (0.54)	Active (0.57)	Active (0.53)	Active (0.54)	Active (0.55)	Active (0.55)
AMES toxicity	No	No	No	No	No	No	No	No	No
Skin sensitisation	No	No	No	No	No	No	No	No	No
hERG I inhibitor	No	No	No	No	No	No	No	No	No
hERG II inhibitor	No	No	No	Yes	No	Yes	No	No	Yes

* Values in parentheses represent the probability; BBB—blood–brain barrier; hERG—human ether-à-go-go-related gene.

**Table 9:** Composition of the investigated polyherbal phytopreparation Tinctura paradentoica^®^ (Institute “Dr Josif Pančić”, Belgrade, Republic of Serbia).

Herbal Ingredient (Biological Source: Plant Species and Family)	Percent (%)
*Calendulae tinctura* (*Calendula officinalis* L., Asteraceae)	15
*Millefolii tinctura* (*Achillea millefolium* L., Asteraceae)	20
*Origani tinctura* (*Origanum vulgare* L., Lamiaceae)	20
*Polygonii tinctura* (*Polygonum aviculare* L., Polygonaceae)	20
*Salviae tinctura* (*Salvia officinalis* L., Lamiaceae)	20
*Tormentillae tinctura* (*Potentilla erecta* (L.) Raeusch., Rosaceae)	5
*Menthae piperitae aetheroleum* (*Mentha × piperita* L., Lamiaceae)	0.02

## Data Availability

The original contributions presented in this study are included in the article/Supplementary Material. Further inquiries can be directed to the corresponding author.

## Supplementary Materials

The following supporting information can be downloaded at https://www.mdpi.com/article/10.3390/ph19030398/s1. Figure S1: Two-dimensional representation of the interaction between chlorogenic acid and amino acids inside human COX-1; Figure S2. Two-dimensional representation of the interaction between gallic acid and amino acids inside human COX-1. Figure S3. Two-dimensional representation of the interaction between hyperoside and amino acids inside human COX-1; Figure S4. Two-dimensional representation of the interaction between isorhamnetin and amino acids inside human COX-1; Figure S5. Two-dimensional representation of the interaction between luteolin and amino acids inside human COX-1; Figure S6. Two-dimensional representation of the interaction between narcissin and amino acids inside human COX-1; Figure S7. Two-dimensional representation of the interaction between rosmarinic acid and amino acids inside human COX-1; Figure S8. Two-dimensional representation of the interaction between rutin and amino acids inside human COX-1; Figure S9. Two-dimensional representation of the interaction between vitexin and amino acids inside human COX-1; Figure S10. Two-dimensional representation of the interaction between chlorogenic acid and amino acids inside human COX-2; Figure S11. Two-dimensional representation of the interaction between gallic acid and amino acids inside human COX-2; Figure S12. Two-dimensional representation of the interaction between hyperoside and amino acids inside human COX-2; Figure S13. Two-dimensional representation of the interaction between isorhamnetin and amino acids inside human COX-2; Figure S14. Two-dimensional representation of the interaction between luteolin and amino acids inside human COX-2; Figure S15. Two-dimensional representation of the interaction between narcissin and amino acids inside human COX-2; Figure S16. Two-dimensional representation of the interaction between rosmarinic acid and amino acids inside human COX-2; Figure S17. Two-dimensional representation of the interaction between rutin and amino acids inside human COX-2; Figure S18. Two-dimensional representation of the interaction between vitexin and amino acids inside human COX-2.

## Abbreviations

The following abbreviations are used in this manuscript:

COX	Cyclooxygenase
GI	Gingival index
PPD	Periodontal pocket depth
SRP	Scaling root planing
DPX	Dibutyl phthalate xylene
HPLC	High-performance liquid chromatography
MVD	Molegro Virtual Docker
