# Supplementary material for: Topical Polyherbal Phytopreparation Reduces Gingival Inflammation: Evidence from a Randomized Controlled Clinical Study Supported by In Silico Analysis
Source: Pharmaceuticals (Basel). 2026 Feb 28;19(3):398. doi: 10.3390/ph19030398 (PMC13029446; doi:10.3390/ph19030398)
Supplement: Supplementary file 1 [file pharmaceuticals-19-00398-s001.zip › pharmaceuticals-4086380-supplementary.pdf]

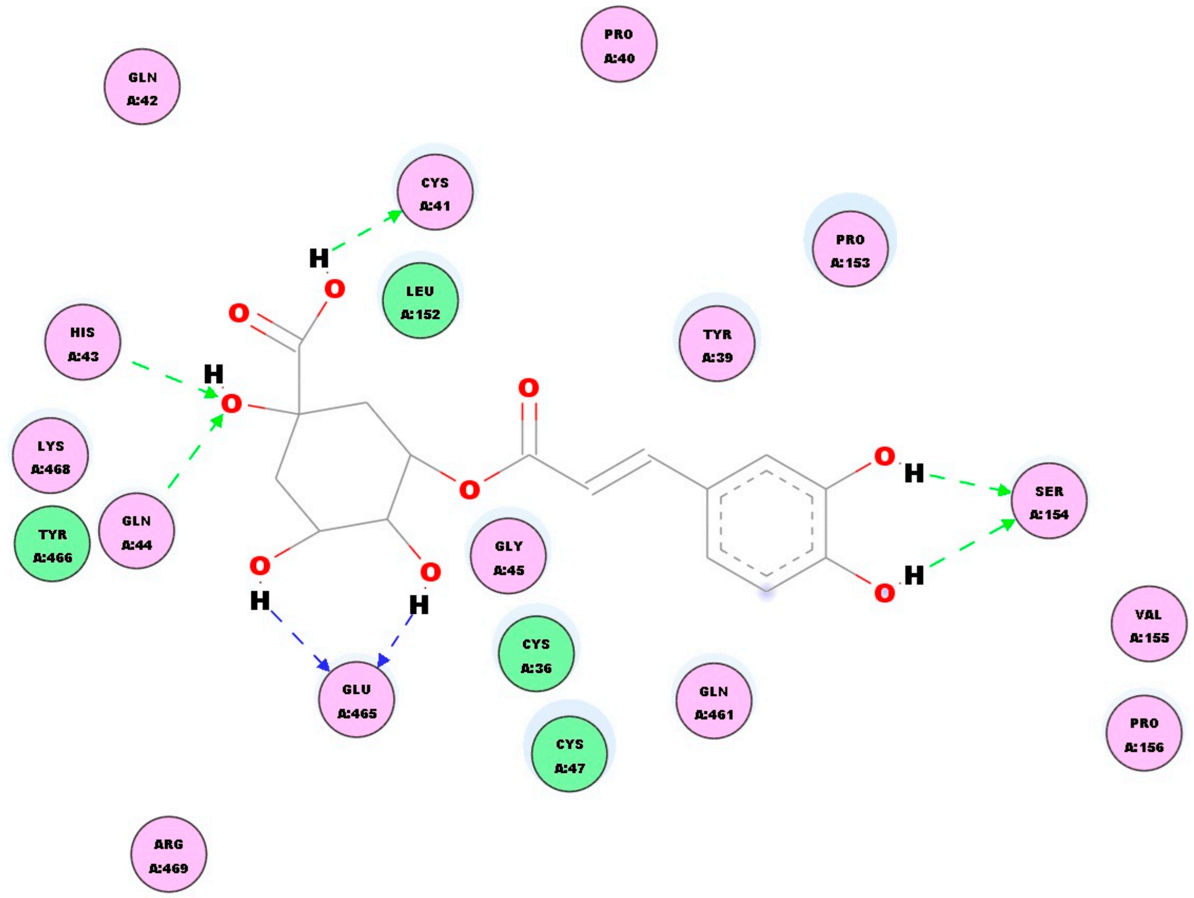

Figure S1. Two-dimensional representation of the interaction between chlorogenic acid and amino acids inside human COX-1.

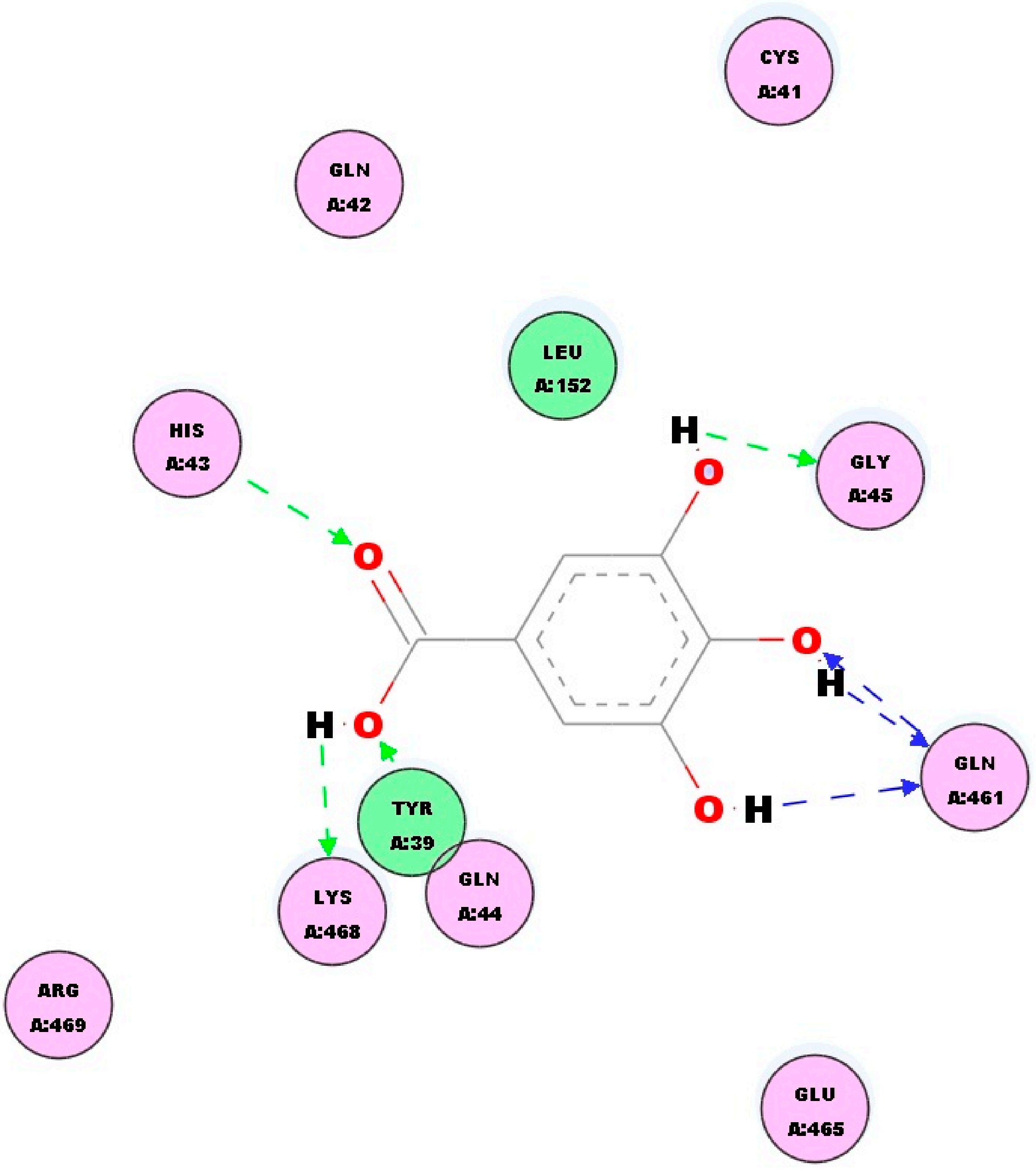

Figure S2. Two-dimensional representation of the interaction between galic acid and amino acids inside human COX-1.

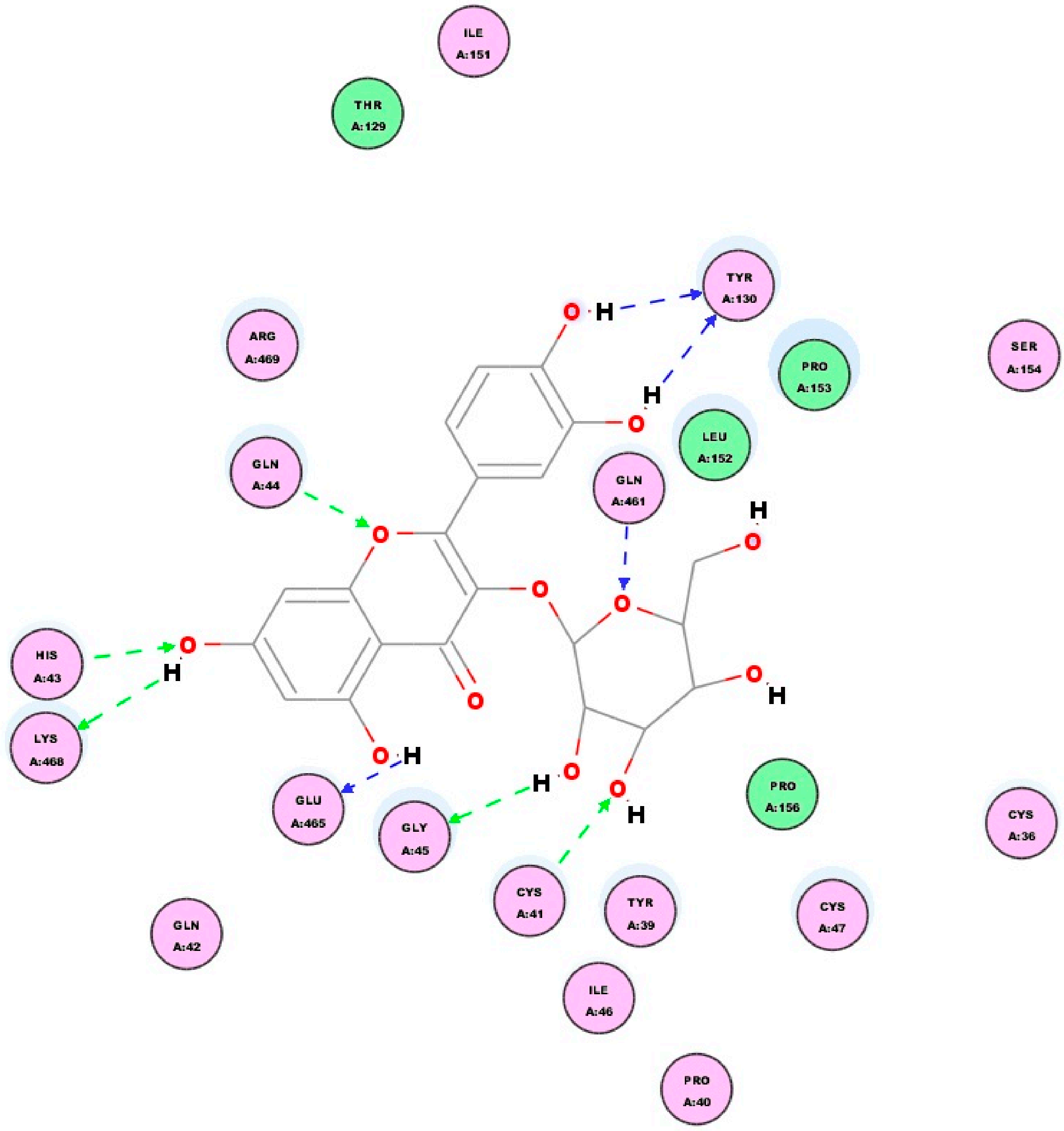

Figure S3. Two-dimensional representation of the interaction between hyperoside and amino acids inside human COX-1.

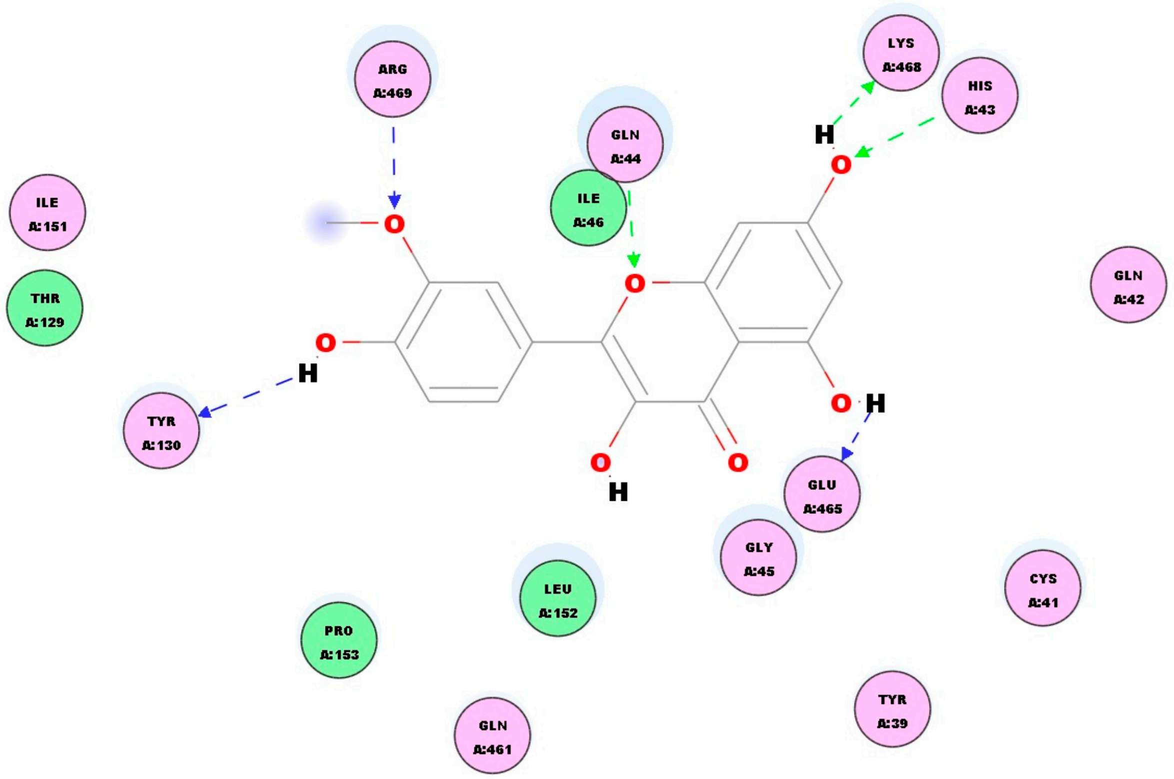

Figure S4. Two-dimensional representation of the interaction between isorhamnetin and amino acids inside human COX-1.

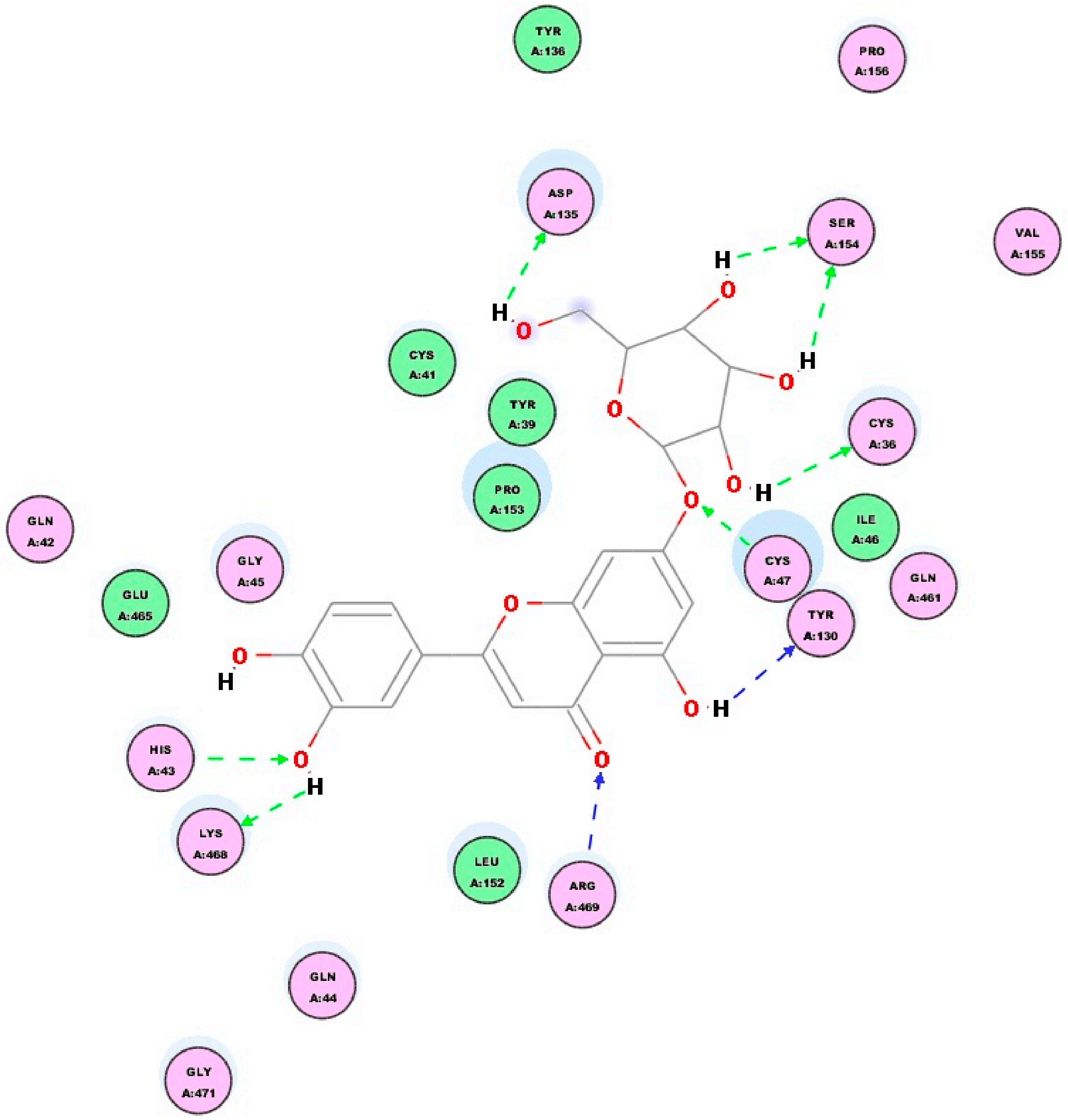

Figure S5. Two-dimensional representation of the interaction between luteolin and amino acids inside human COX-1.

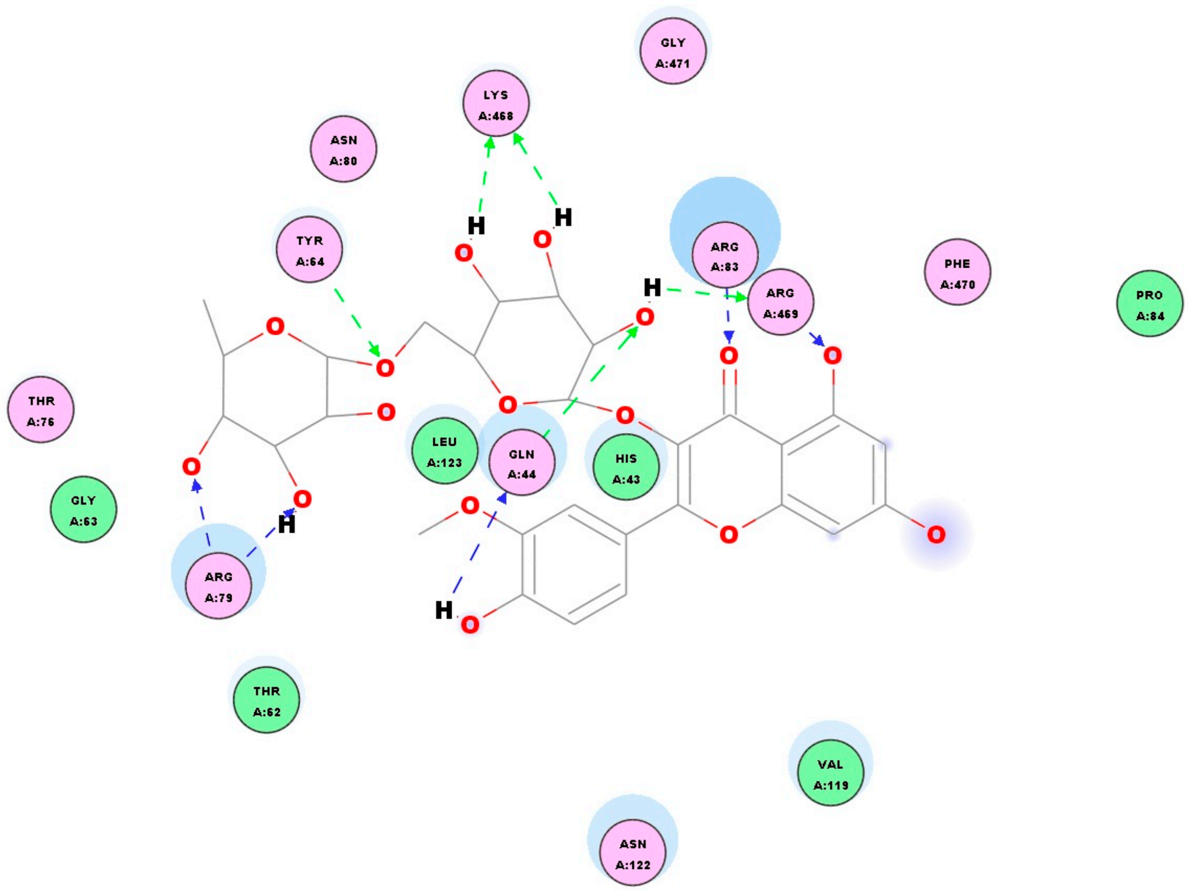

Figure S6. Two-dimensional representation of the interaction between narcissin and amino acids inside human COX-1.

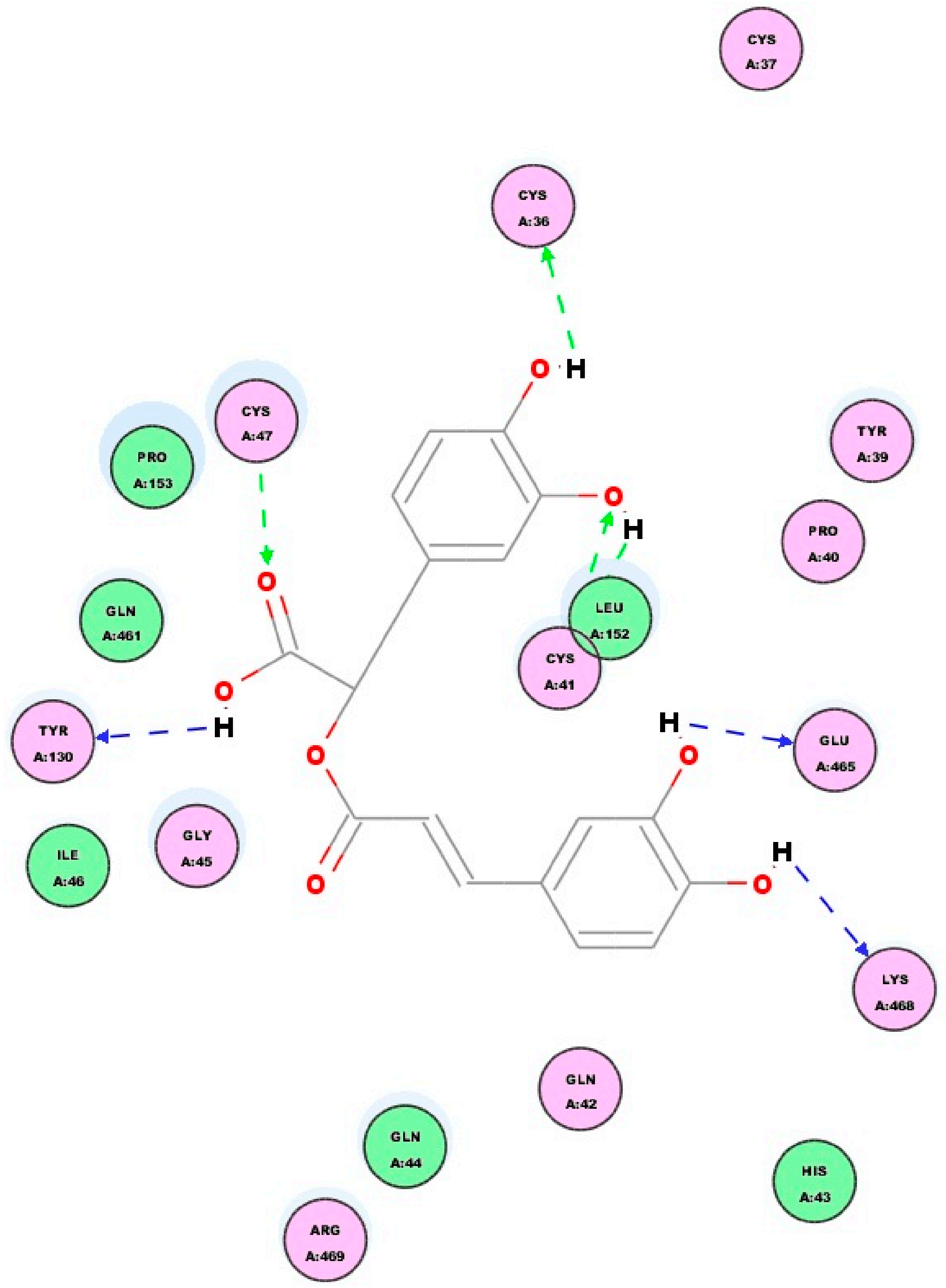

Figure S7. Two-dimensional representation of the interaction between rosmarinic acid and amino acids inside human COX-1.

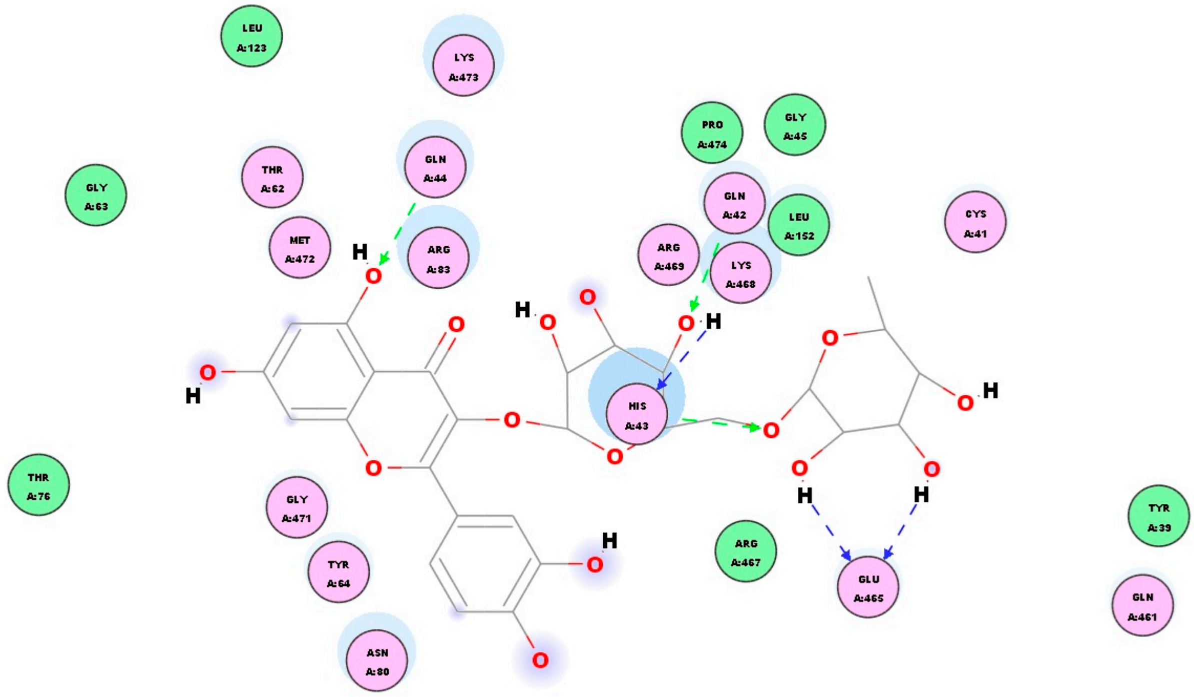

Figure S8. Two-dimensional representation of the interaction between rutin and amino acids inside human COX-1.

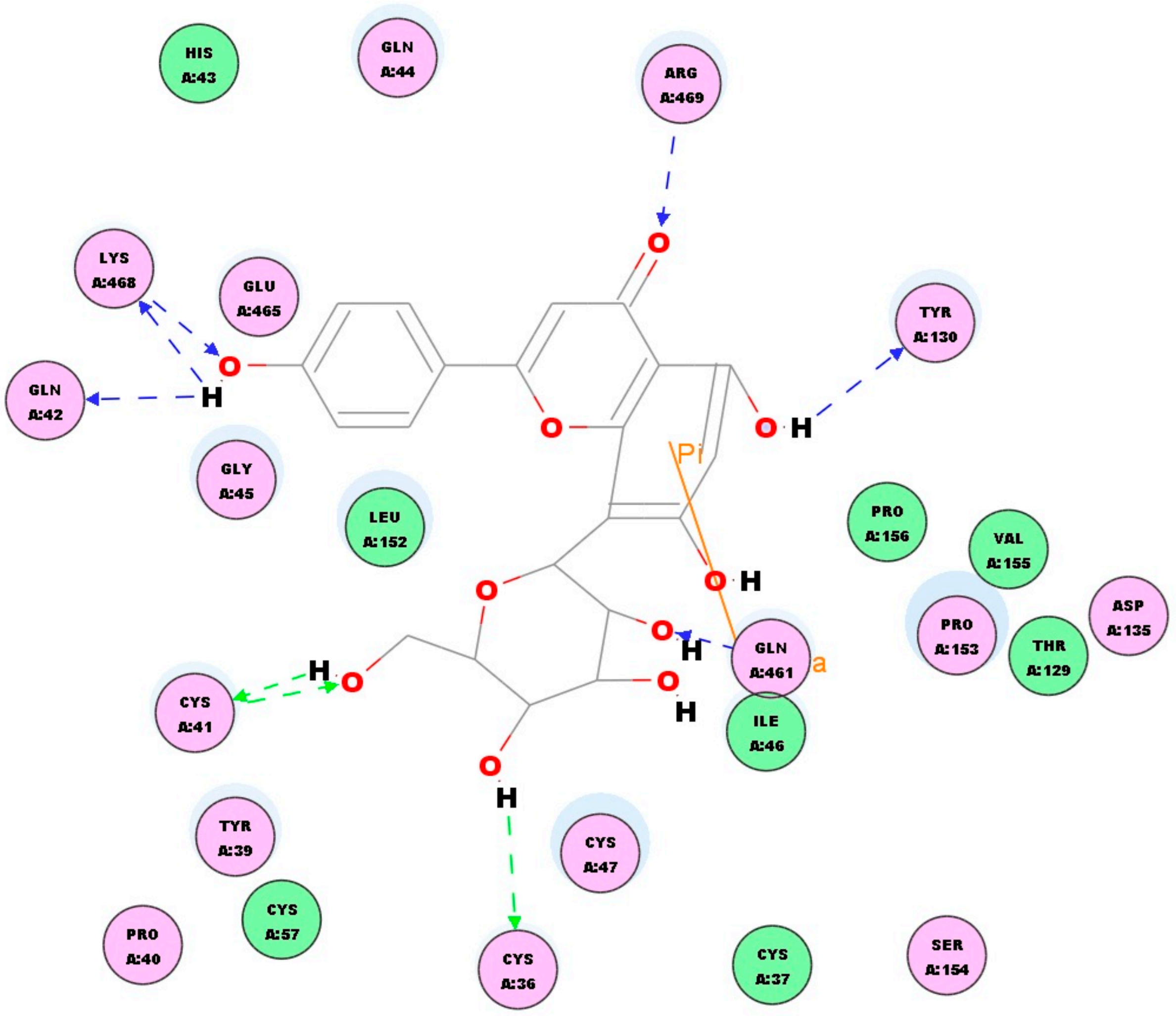

Figure S9. Two-dimensional representation of the interaction between vitexin and amino acids inside human COX-1.

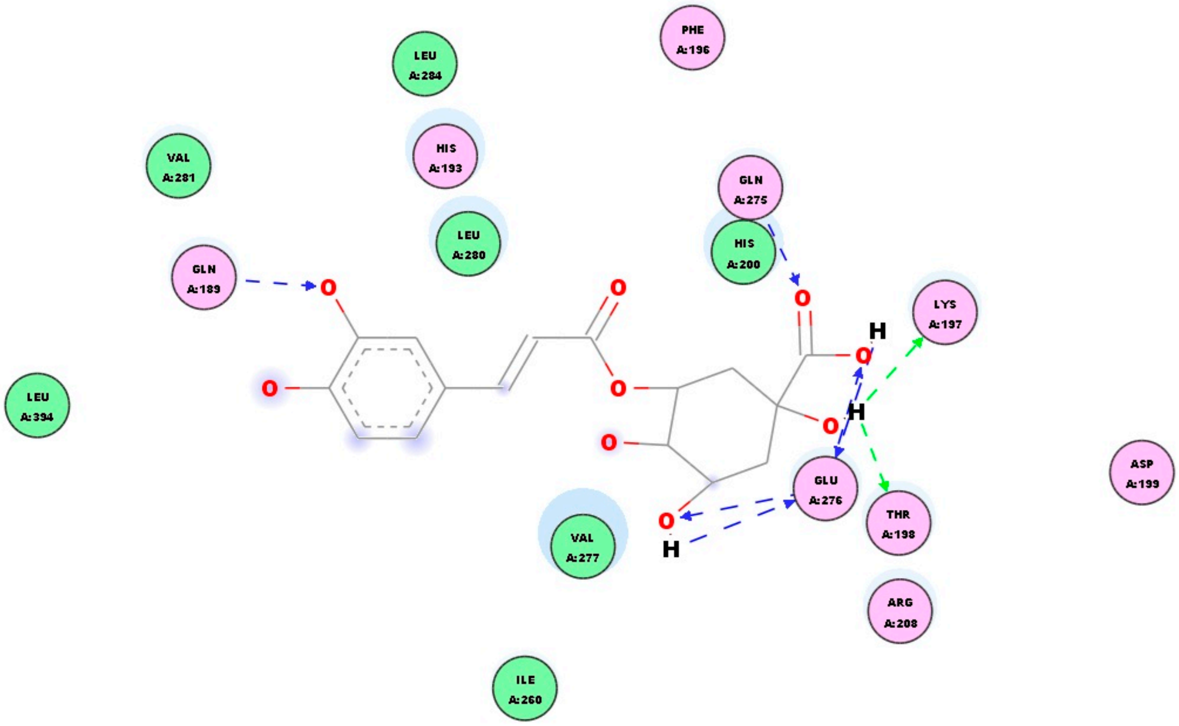

Figure S10. Two-dimensional representation of the interaction between chlorogenic acid and amino acids inside human COX-2.

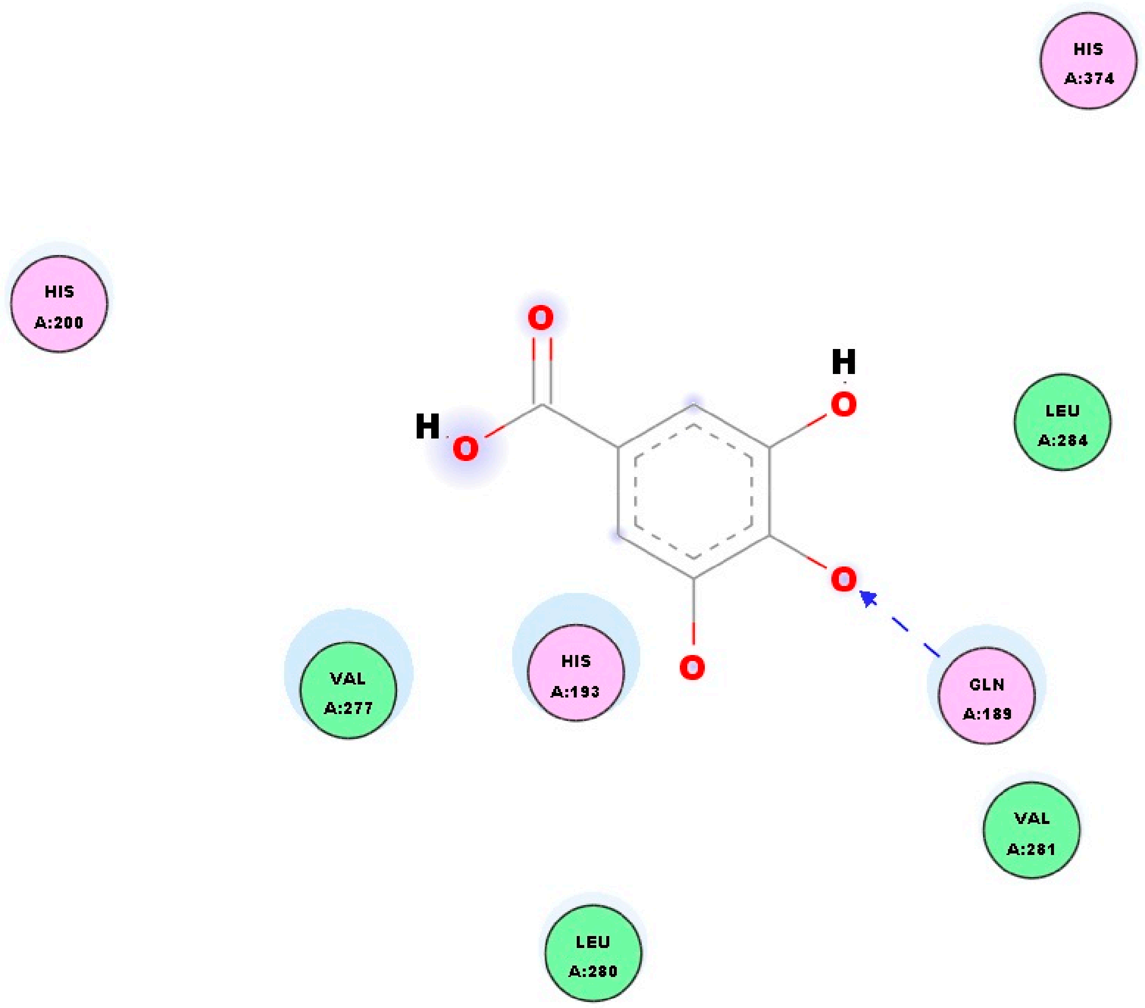

Figure S11. Two-dimensional representation of the interaction between galic acid and amino acids inside human COX-2.

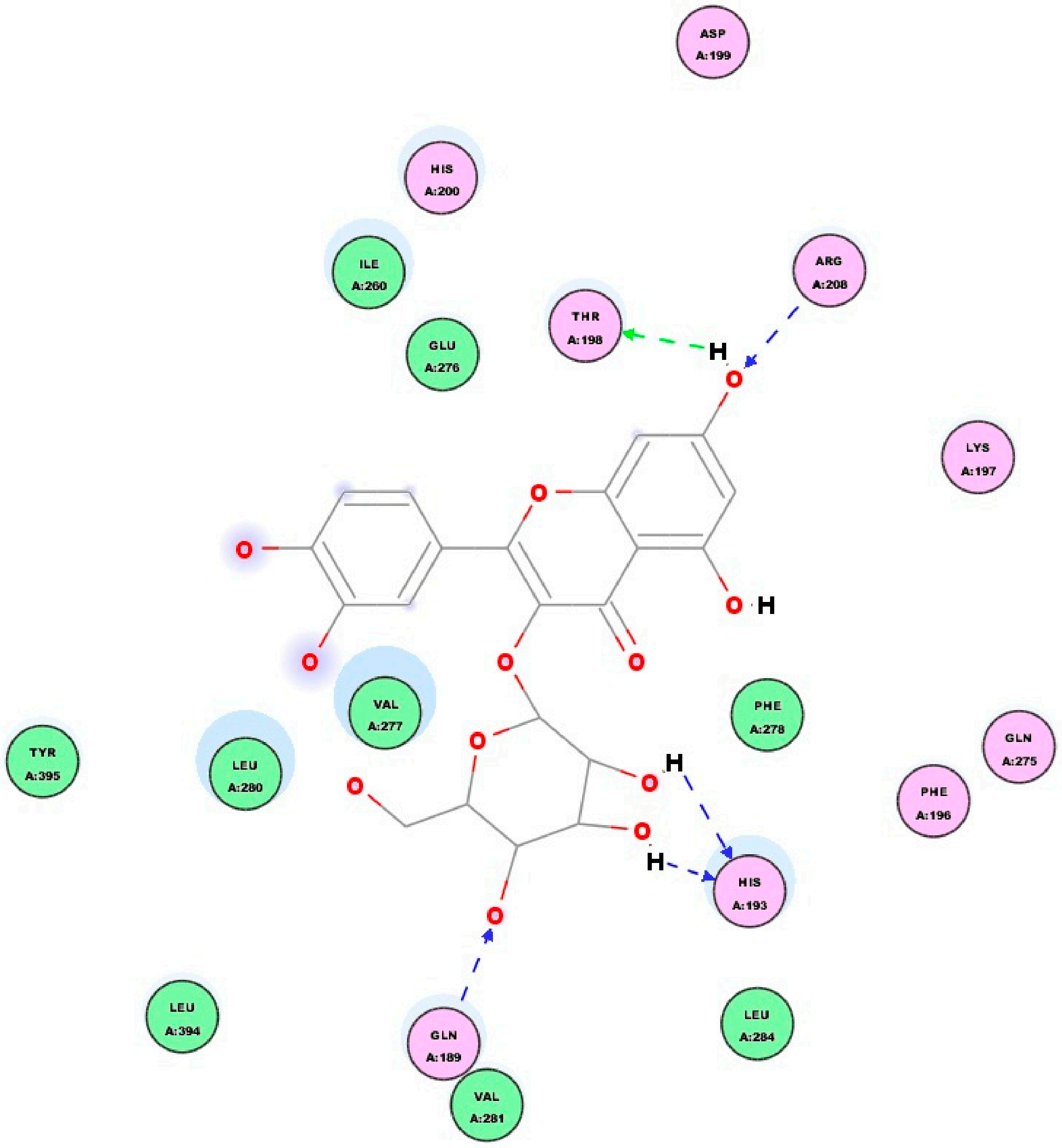

Figure S12. Two-dimensional representation of the interaction between hyperoside and amino acids inside human COX-2.

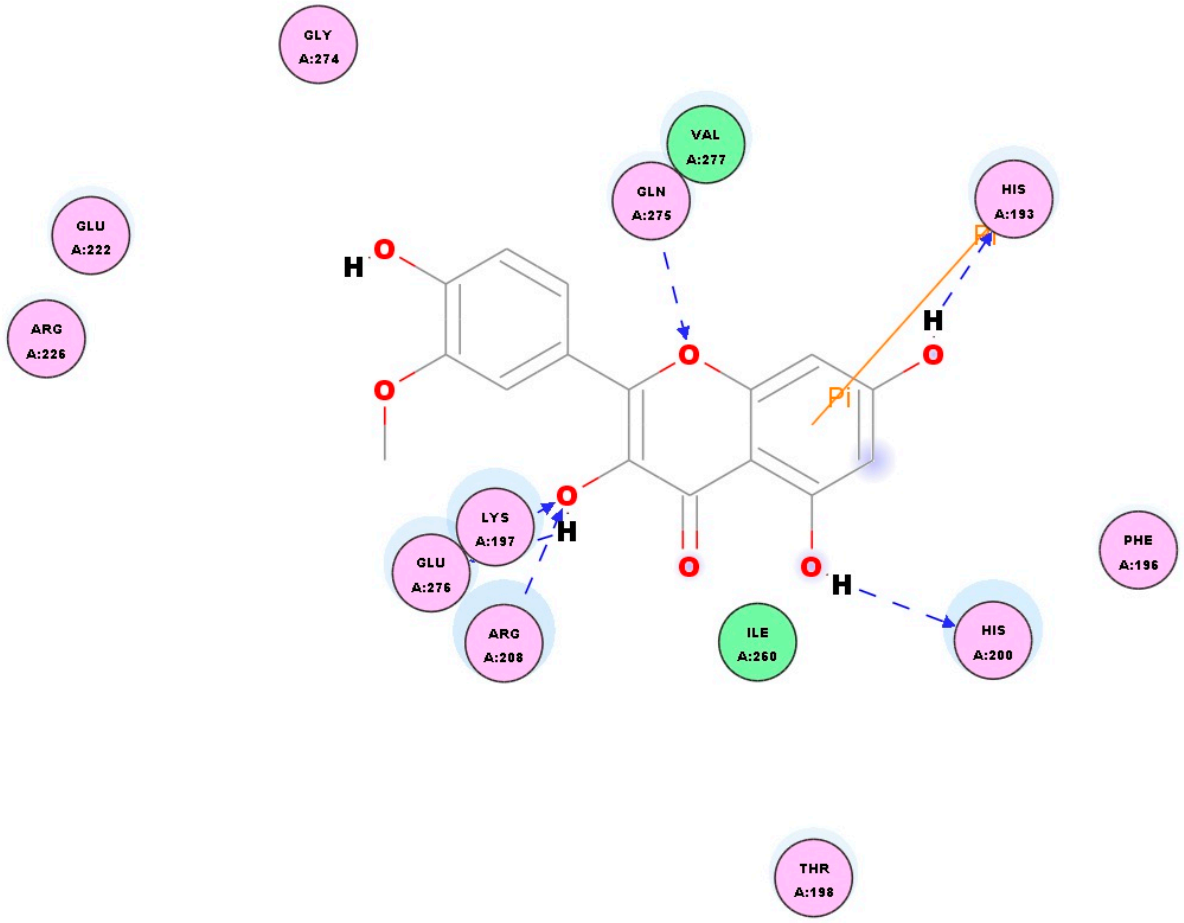

Figure S13. Two-dimensional representation of the interaction between isorhamnetin and amino acids inside human COX-2.

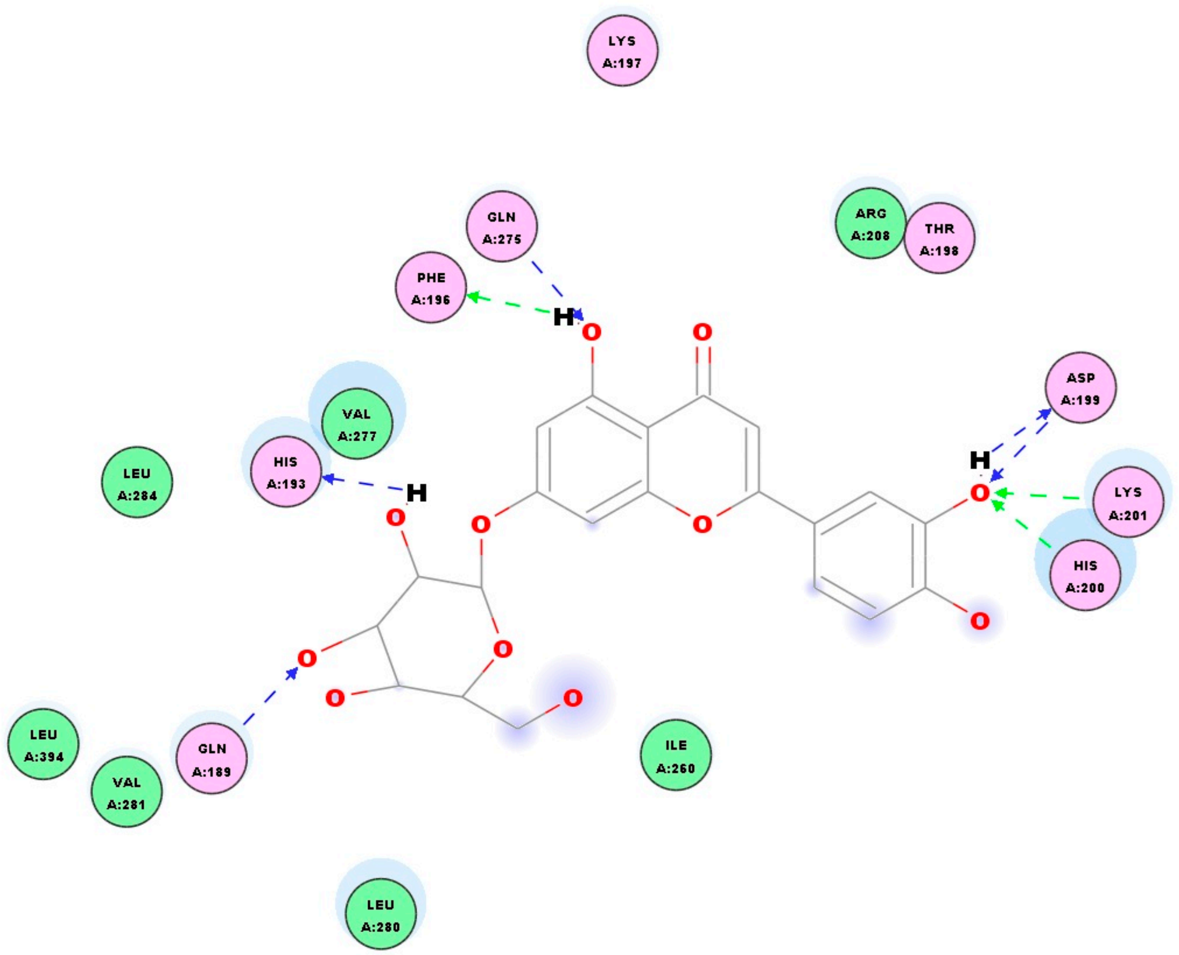

Figure S14. Two-dimensional representation of the interaction between luteolin and amino acids inside human COX-2.

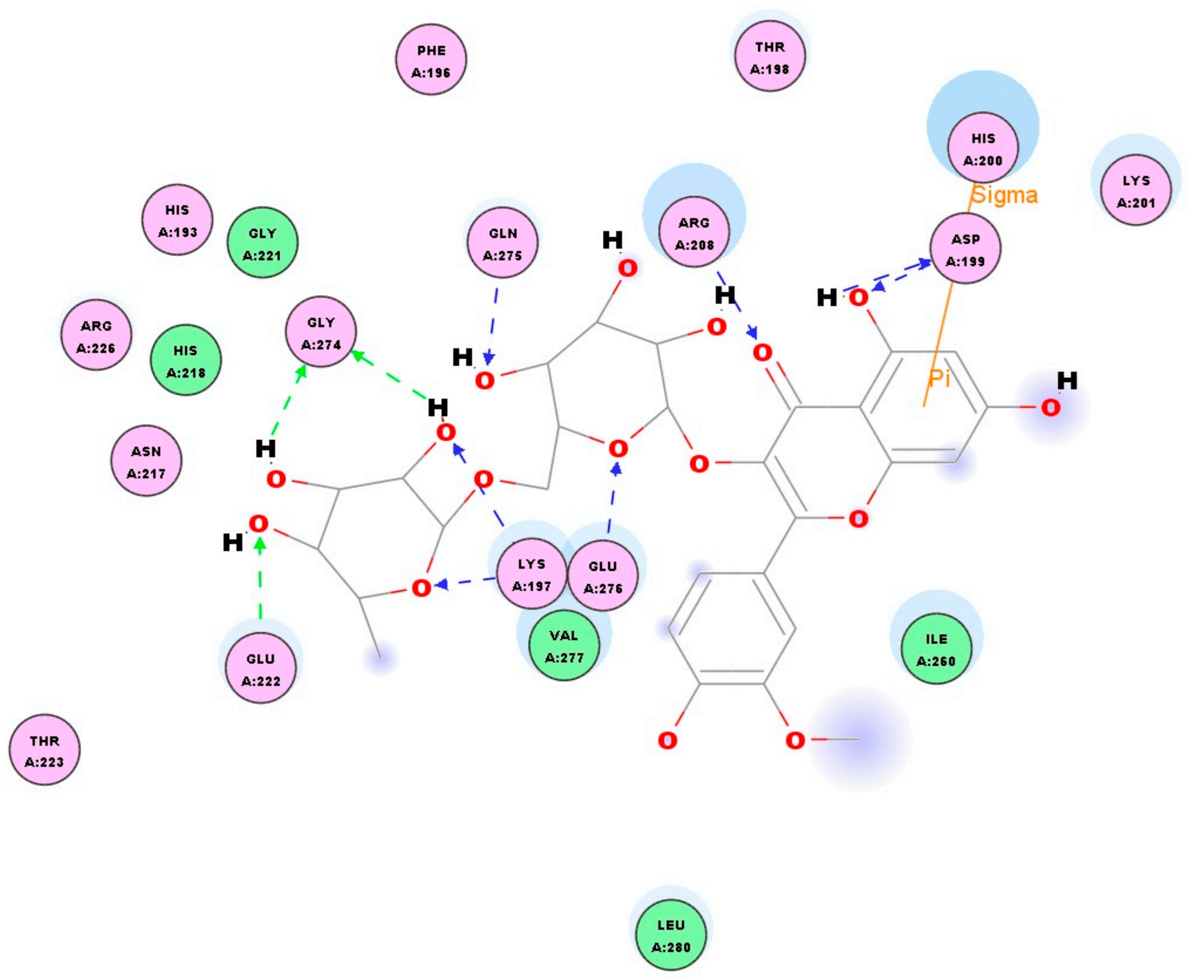

Figure S15. Two-dimensional representation of the interaction between narcissin and amino acids inside human COX-2.

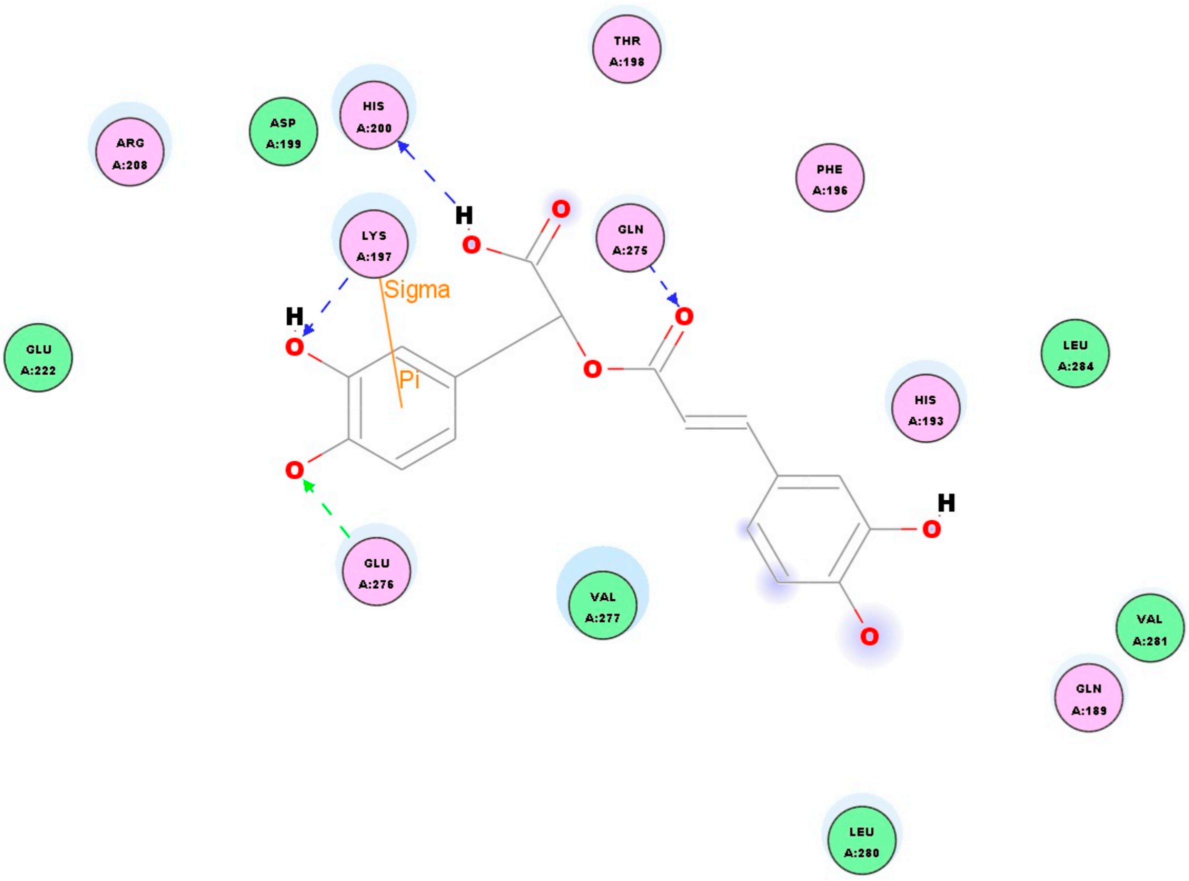

Figure S16. Two-dimensional representation of the interaction between rosmarinic acid and amino acids inside human COX-2.

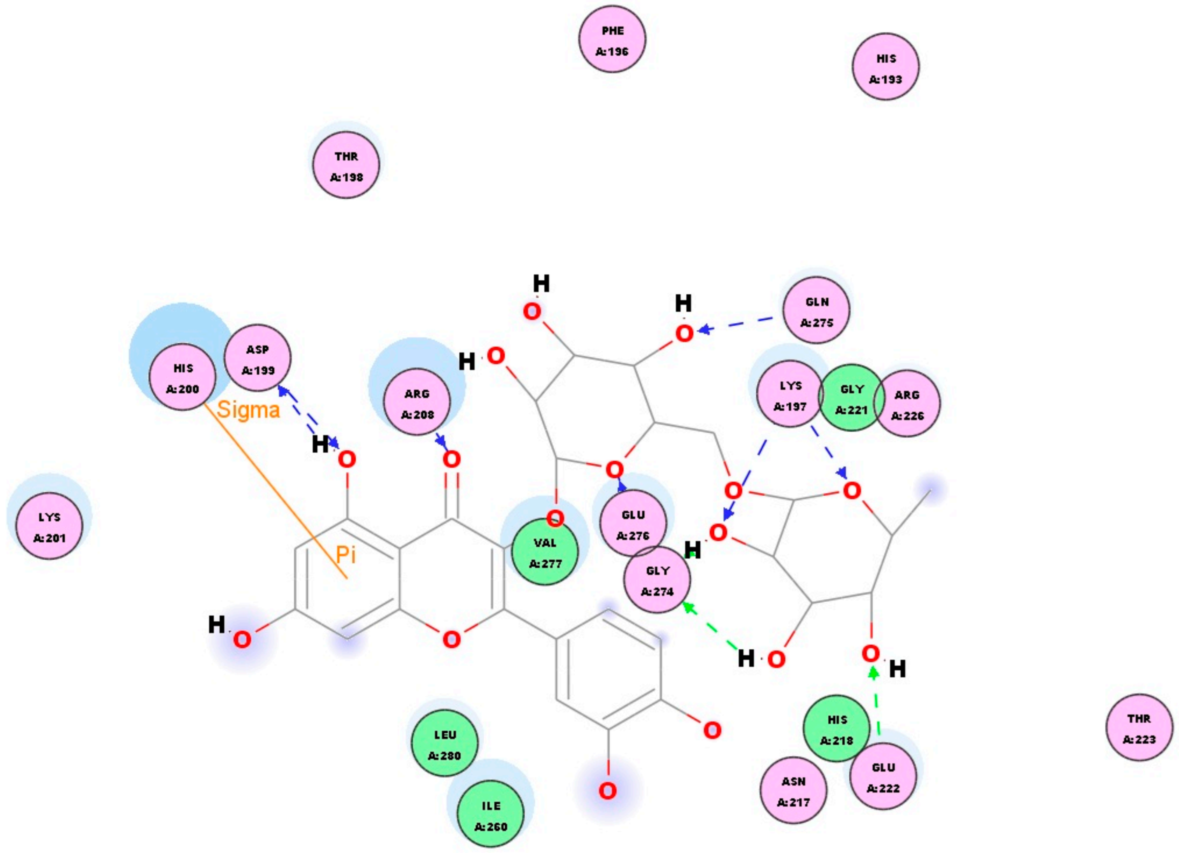

Figure S17. Two-dimensional representation of the interaction between rutin and amino acids inside human COX-2.

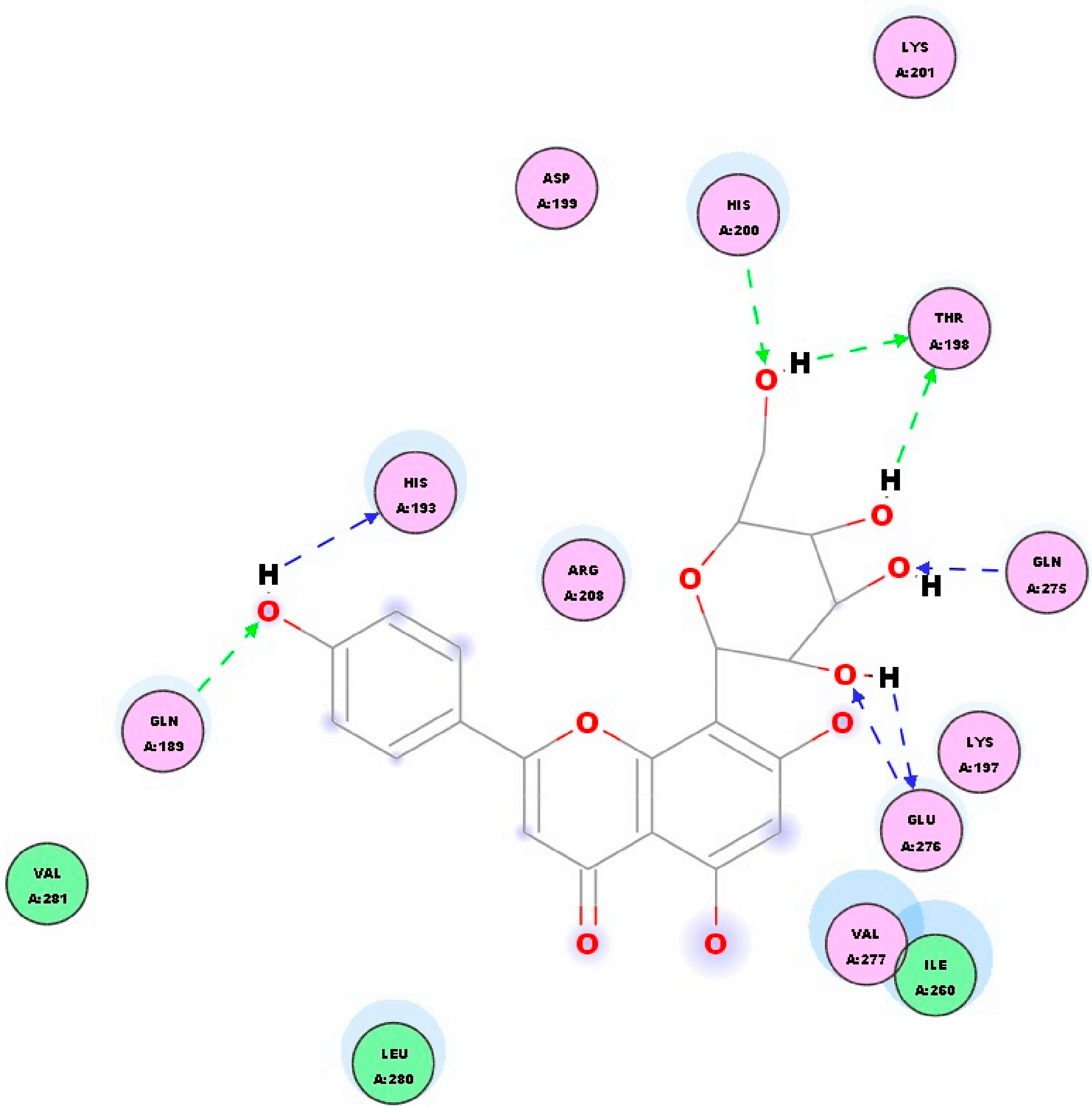

Figure S18. Two-dimensional representation of the interaction between vitexin and amino acids inside human COX-2.
